# Early enterovirus translation deficits extend viral RNA replication and elicit sustained MDA5-directed innate signaling

**DOI:** 10.1128/mbio.01915-23

**Published:** 2023-11-14

**Authors:** Mikhail I. Dobrikov, Elena Y. Dobrikova, Dasean T. Nardone-White, Zachary P. McKay, Michael C. Brown, Matthias Gromeier

**Affiliations:** 1 Department of Neurosurgery, Duke University Medical School, Durham, North Carolina, USA; 2 Department of Molecular Genetics and Microbiology, Duke University Medical School, Durham, North Carolina, USA; Princeton University, Princeton, New Jersey, USA

**Keywords:** poliovirus, MDA5, interferon, IRF3, eIF4G

## Abstract

Enteroviruses (EVs)—positive-sense single-strand RNA viruses of *Picornaviridae*—are among the most common human viral pathogens. Unlike other cytoplasmic viral RNAs (vRNAs), which are detected by RIG-I, sensing of EV RNAs is mediated primarily by MDA5. EVs evolved with rapid, cytotoxic life cycles as a means of host cell interference, ostensibly to suppress MDA5-orchestrated antiviral type-I interferon (IFN) responses. At the core of this strategy are viral 2A protease (2A^pro^)-directed cleavage of the eukaryotic initiation factor (eIF)4G—the central translation initiation scaffold and ribosome adaptor—and degradation of nuclear pores. EV MDA5 agonism has an intriguing innate footprint, characterized by polar TBK1-IFN regulatory factor 3 (IRF3) signaling, which fuels sustained type-I IFN release to provide context for antitumor CD8^+^ T-cell priming after *in situ* cancer vaccination. Here we compared EV-host interactions between wild-type EVs and the highly attenuated recombinant polio:rhinovirus chimera and cancer immunotherapy agent (PVSRIPO) to test how targeted inhibition of discrete steps in the viral life cycle affects the innate antiviral response. Our investigations demonstrate that sustained MDA5-TBK1-IRF3 signaling elicited by PVSRIPO is the result of inefficient immediate 2A^pro^-directed eIF4G degradation, while wild-type EVs counter host innate type-I IFN defenses via rapid eIF4G cleavage. The MDA5-directed innate signature of profoundly attenuated EVs may provide efficient innate immune-stimulatory assets, for example, in *in situ* cancer vaccination strategies.

**IMPORTANCE:**

Multiple pattern recognition receptors sense vRNAs and initiate downstream innate signaling: endosomal Toll-like receptors (TLRs) 3, 7, and 8 and cytoplasmic RIG-I-like receptors (RLRs) RIG-I, and MDA5. They engage distinct signaling scaffolds: mitochondrial antiviral signaling protein (RLR), MyD88, and TLR-adaptor interacting with SLC15A4 on the lysosome (TLR7 and TLR8) and toll/IL-1R domain-containing adaptor inducing IFN (TLR3). By virtue of their unusual vRNA structure and direct host cell entry path, the innate response to EVs uniquely is orchestrated by MDA5. We reported that PVSRIPO’s profound attenuation and loss of cytopathogenicity triggers MDA5-directed polar TBK1-IRF3 signaling that generates priming of polyfunctional antitumor CD8^+^ T-cell responses and durable antitumor surveillance *in vivo*. Here we unraveled EV-host relations that control suppression of host type-I IFN responses and show that PVSRIPO’s deficient immediate host eIF4G cleavage generates unopposed MDA5-directed downstream signaling cascades resulting in sustained type-I IFN release.

## INTRODUCTION

Type-I interferons (IFNs) are the primordial defensive bulwark against RNA viruses because of their ability to induce IFN-stimulated genes and pleiotropic effects on innate and adaptive immunity. Most nucleated mammalian cells express the cytoplasmic RNA virus pattern recognition receptors RIG-I and MDA5 and mount antiviral IFN defenses upon sensing viral RNA (vRNA) in the cytoplasm. Adapting to this challenge, human pathogenic RNA viruses evolved with sophisticated ploys to prevent, intercept, or counteract type-I IFN signaling at every conceivable level.

Enterovirus (EV)-host innate relationships are unique among human pathogenic RNA viruses. Sensing of EV RNA is orchestrated principally by MDA5 rather than RIG-I, which detects 5′ppp ends in vRNAs of most RNA virus families (1–3). EV RNA 5′ ends are covalently bound to the genome-linked protein VPg (4), which precludes RIG-I sensing and canonical translation initiation via eukaryotic initiation factor (eIF)4E binding to a 5′ 7-methyl-guanosine (m^7^G) “cap” (5). EVs prevent innate antiviral IFN release at least in part through drastic, cytopathogenic interference with host cell protein synthesis via 2A protease (2A^pro^)-directed cleavage of the translation initiation scaffold eIF4G. Two eIF4G isoforms (eIF4G1 and eIF4G2) enable m^7^G-cap-dependent translation initiation by bridging mRNA 5′ ends to 40S ribosomal subunits via binding to eIF4E and eIF3 (6). eIF4G1 and eIF4G2 are cleaved by EVs (7), inhibiting cap-dependent host protein synthesis by separating the N-terminal eIF4E-binding sites from the C-terminal eIF3-binding sites (8). This does not affect viral translation, as the EV internal ribosomal entry sites (IRESs) recruit ribosomes by binding eIF4G, or the C-terminal eIF4G cleavage fragment generated by 2A^pro^-directed cleavage, without eIF4E involvement (9).

We previously reported that polio:rhinovirus chimera and cancer immunotherapy agent (PVSRIPO), the live attenuated type-1 poliovirus (Sabin) vaccine replicating under control of a human rhinovirus (HRV) type 2 IRES (10), bolsters tumor immune surveillance due to sustained type-I IFN dominant inflammation in the tumor microenvironment (11, 12). In this work, we compared innate antiviral immunity to PVSRIPO with the (wild-type) cytopathogenic EV coxsackievirus A21 (CAV21) and human rhinovirus type 16 (HRV16). We tested the impact of targeted inhibitors of specific steps in the EV life cycle on the host innate antiviral response. We show that in wild-type EV infection, incipient viral translation of incoming vRNA causes 2A^pro^-directed eIF4G1 cleavage and prevents MDA5 downstream signaling and early type-I IFN synthesis, thus intercepting the transcriptional network driving sustained IFN-α release. Our observations indicate that PVSRIPO’s encumbered early host interference resulting in an extreme level of attenuation is optimal for sustained type-I IFN release required for effective *in situ* cancer vaccination.

## RESULTS

### Failure of PVSRIPO to induce immediate eIF4G cleavage is associated with inefficient early translation

Ever since transitioning to immortal HeLa cell lines in the 1950s (13), poliovirus (PV) research has been dominated by this *in vitro* model. In lieu of wild-type PV, we employ CAV21—a member of the “C-CAVs” (receptor: intercellular adhesion molecule 1 [ICAM1])—which, together with the PVs, are part of the HEV-C species of *Enteroviridae*. The C-CAVs likely gave rise to the PVs (14); CAV21 causes paralytic poliomyelitis in human ICAM1-transgenic mice (15), analogous to the PVs in human CD155-transgenic mice (16).

**Fig 1:**
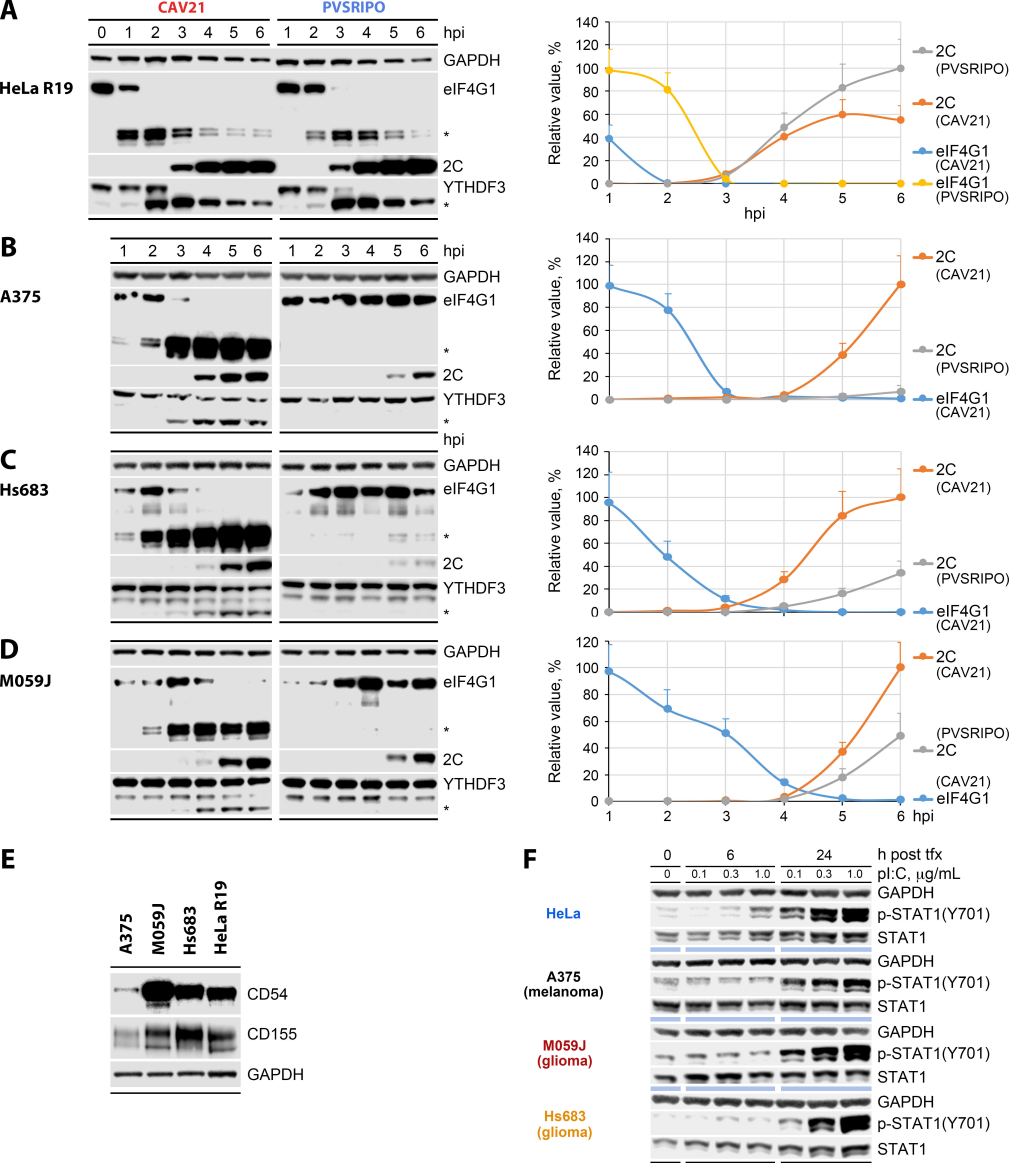
Short-term time course of PVSRIPO and CAV21 infection in a panel of in vitro tumor models (multiplicity of infection [MOI], 10). (A–D) HeLa R19 (A), A375 (B), Hs683 (C), and M059J (D) cells were infected with PVSRIPO or CAV21. Lysates collected at the indicated hpi were analyzed by immunoblot for viral translation (2C) and 2A^pro^-directed cleavages (eIF4G, YTHDF3; asterisks indicate eIF4G/YTHDF3 cleavage products). All assays were performed at least three times, and representative immunoblots are shown. The panels in the right column depict quantification of protein levels (% max. detected, normalized to the glyceraldehyde-3-phosphate dehydrogenase (GAPDH) loading control; means ± standard error of the mean), representing the average values from three independent series. (E) Expression of poliovirus receptor PVR (CD155) and CAV21 receptor ICAM1 (CD54) in the panel. (F) P-STAT1(Y701) response to transfection of increasing concentrations of poly(I:C) in the cell line panel. Cell lysates were collected at the indicated intervals and tested for p-STAT1(Y701)/STAT1 by immunoblot.

In CAV21-infected HeLa cells, eIF4G1 cleavage is immediate (Fig. 1A). Because the eIF4G1:2 ratio in cells is ~10:1 (17), we only probe for eIF4G1 (referred to as eIF4G from hereon). Cleavage is near complete prior to “processive” viral translation, readily detected by immunoblot, which occurs after vRNA replication begins. At 2 hpi, intact eIF4G was reduced to <1% of native levels with viral 2C levels at <1% of the maximum (Fig. 1A). PVSRIPO yielded delayed eIF4G cleavage in HeLa cells compared to CAV21 (at 2 hpi, remaining intact eIF4G was ~80% vs <1% for CAV21) mirrored by YTHDF3, another 2A^pro^-cleaved host protein (Fig. 1A) (18). While eIF4G cleavage was faster in CAV21-infected HeLa cells, the levels of eIF4G cleavage observed with PVSRIPO and CAV21 are invariably cytopathogenic (11).

Yet, HeLa cells do not reliably model PVSRIPO relations with authentic non-malignant PV host cells, e.g., myeloid cells. Probing PVSRIPO susceptibility in monocyte-derived dendritic cells (DCs), monocyte-derived macrophages (MDMs), or *ex vivo* malignant glioma slices revealed a failure to execute eIF4G cleavage with absent cytopathogenicity (11, 12, 19, 20). We reported PVSRIPO cytotoxicity with eIF4G cleavage in many *in vitro* tumor models (10, 11), including after IFN-α pre-treatment of cells (21). We identified a panel of tumor models, A375 melanoma and Hs683/M059J malignant glioma cell lines, that mirrors loss of cytopathogenicity/eIF4G cleavage activity observed in slice culture assays (Fig. 1B through D). Exemplifying the cytopathogenic host relations of wild-type EVs, CAV21 infection yielded efficient eIF4G cleavage in our panel at par with HeLa cells (Fig. 1A through D). The CAV21:PVSRIPO differential is reflected in puromycylation assays of protein synthesis activity, documenting suppression of host translation in CAV21- but not PVSRIPO-infected A375 cells (Fig. S1).

The cell line panel expresses the PV (CD155) and CAV21 (ICAM1, CD54) receptors at levels mediating uniform susceptibility to infection (Fig. 1E). Transfection of high-molecular weight (~1.5 to 8.0 kB) poly(I:C)—to engage MDA5 (22)—revealed similar p-STAT1(Y701) responsiveness across the panel (Fig. 1F). Thus, variable PVSRIPO cytopathogenicity in the panel is not due to differences in entry or innate sensing/signaling. Rather, it may be due to cell line-specific conditions for IRES-mediated translation of incoming vRNA (driving 2A^pro^-directed cleavages), evident as substantially elevated PVSRIPO 2C at 3–6 hpi in HeLa compared to A375, Hs683, or M059J cells (Fig. 1A through D).

### EV-directed eIF4G cleavage prevents IRF7 induction/activation

**Fig 2:**
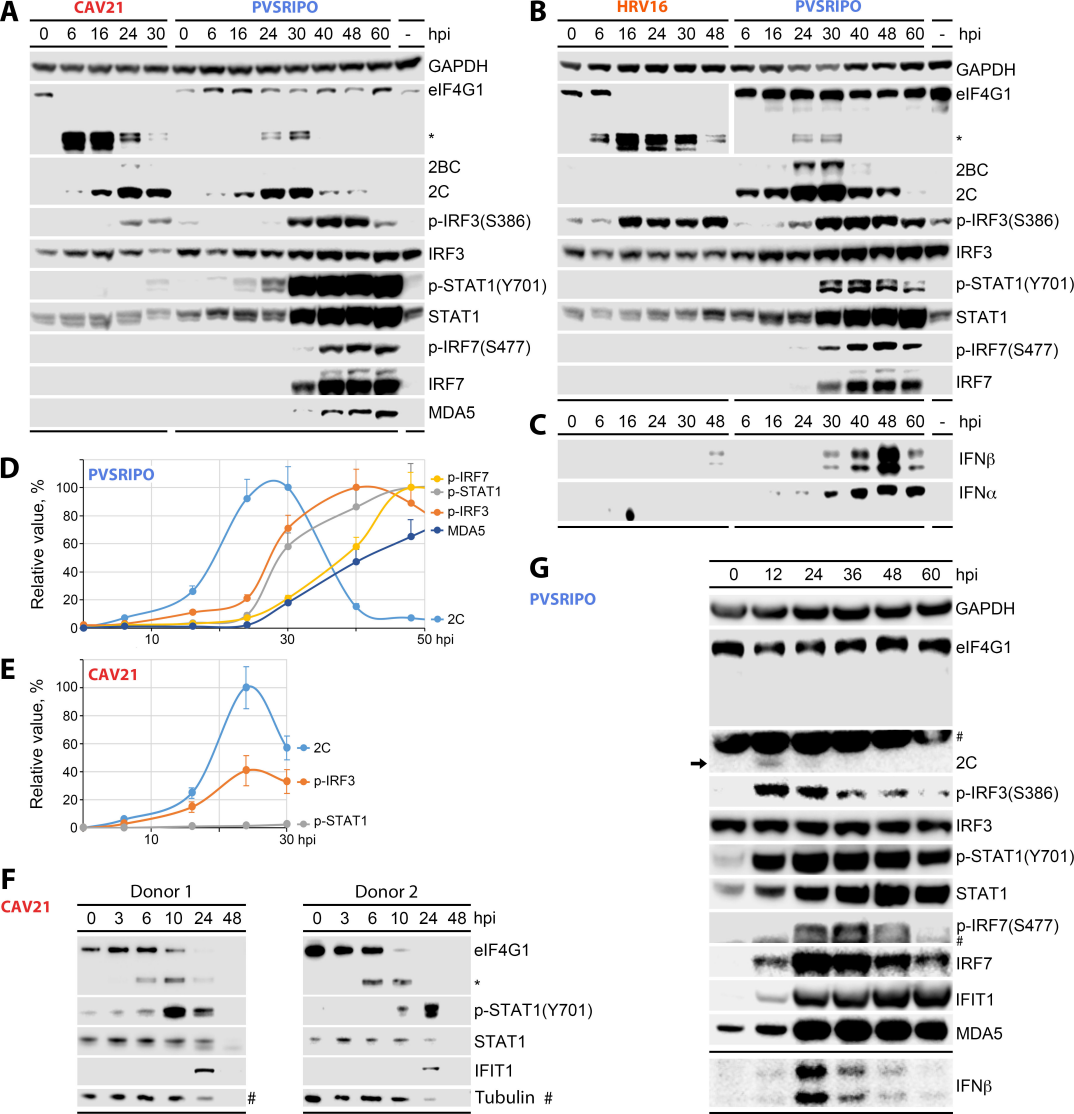
Long-term time course of EV infection in A375- and myeloid antigen-presenting cells. (**A and B**) A375 cells were infected with CAV21 or PVSRIPO (MOI, 10 [**A**]) and with HRV16 or PVSRIPO (MOI, 10 [**B**]). HRV16 infections were carried out at 33°C. Lysates were analyzed by immunoblot for viral translation (eIF4G cleavage, 2C/2BC); TBK1 activation [p-IRF3(S386)/IRF3]; and type-I IFN signaling [p-STAT1(Y701)/STAT1, p-IRF7(S477)/IRF7, MDA5] over time (*eIF4G1 cleavage products). (**C**) IFN-α/IFN-β immunoblots. (**D and E**) Expression levels of viral 2C and the indicated (phospho)-proteins in PVSRIPO-infected cells (**D**) or in CAV21-infected cells (**E**) were quantified. The values represent the average abundance of proteins in three independent series (% max. detected, normalized to GAPDH; mean ± standard error of the mean). The assays were performed in at least three independent series; representative results are shown. (**F and G**) Long-term time course of CAV21 (MOI, 10 [**F**]) and PVSRIPO (MOI, 10 [**G**]) infection in human monocyte-derived antigen-presenting cells. Lysates collected at the indicated intervals were analyzed by immunoblot for the parameters shown. Cells from two distinct donors were tested with CAV21 infection; #, loss of signal with the tubulin loading control (and all other proteins) at 24–48 hpi are due to rampant cytolysis (**F**). PVSRIPO infection of MDMs (**G**) was analyzed by immunoblot of samples collected at the indicated intervals. Cells from three distinct donors were tested in five independent series; representative results are shown with samples from one donor (the arrow indicates weak signal for viral 2C; # indicates prominent non-specific background bands).

vRNA sensing by MDA5/RIG-I initiates a signaling cascade that drives transcriptional programs leading to type-I IFN (IFN-α/IFN-β) production, coordinated in part by sequential and overlapping activation of IFN regulatory factors (IRF)3 and IRF7. IRF3/IRF7 control *IFNA*/*B* transcription yielding specific temporal release patterns of IFN-β and the 12 IFN-α isoforms (23). To unravel the relation of cytopathogenic profile with the antiviral type-I IFN response, we compared PVSRIPO to EVs on the opposite ends of a spectrum of intrinsic cytopathogenic potential: CAV21, exemplifying the cytotoxic HEV-C (Fig. 2A) and HRV16 (Fig. 2B), which both use ICAM1 for host cell entry. HRV16 has a naturally non-cytopathogenic phenotype in human airway epithelial cells, its principal human host target cells (24). Viruses were subjected to long-range kinetic analyses of viral translation/cytopathogenicity and the innate host response in A375 cells (Fig. 2A through E).

In PVSRIPO-infected A375 cells, biphasic IRF3/IRF7 activation as described by Sato et al. (25) unfolded (Fig. 2A through C), evident upon quantitative assessment of the kinetics of the innate signaling response (Fig. 2D). Activating phosphorylation of (constitutively expressed) IRF3 led to early IFN-α/IFN-β release, robust STAT1(Y701) phosphorylation, and sustained type-I IFN-mediated IRF7 induction/activation (Fig. 2D). In contrast, CAV21-infected cells displayed modest, “futile” IRF3(S386) phosphorylation, ie., IRF3 phosphorylation/activation that did not yield p-STAT-, IRF7 induction/phosphorylation, or type-I IFN-mediated induction of MDA5 (Fig. 2A and E). Since CAV21-infected cells exhibited signs of overt cytopathogenicity after 24 hpi, blunted p-IRF3 and failing IRF7 activation likely are the result of eIF4G cleavage-triggered damage. Surprisingly, HRV16 infection also failed to trigger p-STAT1(Y701)/IRF7(S477) and STAT1/IRF7 induction (Fig. 2B). In HRV16-infected cells, compared to CAV21, eIF4G cleavage was delayed, and p-IRF3(S386) levels resembled those seen with PVSRIPO (Fig. 2B). Yet, as with CAV21, p-IRF3 downstream signaling events did not occur (Fig. 2B). Thus, HRV16’s delayed—but complete—eIF4G cleavage is sufficient to mitigate IRF7 engagement and thwart sustained type-I IFN responses.

PVSRIPO/HRV16 innate signaling and IRF3/IRF7 activation profiles were reflected in IFN-α/IFN-β expression patterns (Fig. 2C). PVSRIPO induced robust, sustained IFN-α/IFN-β synthesis corresponding to the IRF3/IRF7 activation sequence described above. IFN-β peaked at ~48 hpi. Particularly noteworthy is sustained IFN-α production, which—similar to p-IRF7/MDA5 induction—peaked on or after 60 hpi (Fig. 2C). This is consistent with PVSRIPO’s innate imprint in human primary explant systems, eg., DCs/MDMs or in glioblastoma patient *ex vivo* tumor slices (12). In accordance with its cytopathogenic profile with complete eIF4G cleavage and thwarted IRF7 activation, HRV16 only yielded barely detectable IFN-β (48 hpi), possibly reflecting intact IRF3 activation (Fig. 2B). IFN-α production was not detected at any study interval (Fig. 2C).

PVSRIPO translation (2C) peaked at ~27 hpi, but maximal p-IRF3 levels were only reached until much later, at ~42 hpi (Fig. 2D). All signaling events downstream of p-IRF3, e.g., p-STAT1(Y701), IRF7 induction/S477 phosphorylation, and MDA5 induction, reached their peak on/after 60 hpi (Fig. 2D). Thus, the hallmarks of the PVSRIPO-induced type-I IFN defense are a signaling response that is detached from the kinetics of viral translation and that is characterized by a multistep IRF3-IRF7 phosphorylation/induction cascade producing sustained IFN-α release. This contrasts with the abrogated innate inflammatory response to CAV21, where viral translation and p-IRF3 occurred earlier (24 hpi) and in parallel (Fig. 2E) and where type-I IFN synthesis is absent.

A375 cells are malignant; therefore, we validated CAV21 vs PVSRIPO innate signatures in true, non-transformed PV hosts. We used primary monocyte-derived antigen-presenting cells, authentic PV targets in humans/old-world primates (26). Similar to the wild-type PV reported earlier (27), CAV21 produced complete eIF4G cleavage and pronounced cytopathogenicity in such hosts (Fig. 2F). As in A375 cells, this was accompanied by a blunted type-I IFN response [p-STAT1(Y701), Fig. 2F]. In MDMs infected with PVSRIPO (MOI, 10) (Fig. 2G), viral translation was barely detectable at 12 and 24 hpi (Fig. 2G), consistent with prior findings (12). As in A375/M059J/Hs683 cells, in contrast to wild-type PV (27) and CAV21 (Fig. 2F), PVSRIPO does not cleave eIF4G in myeloid cells (Fig. 2G) (11, 12). Infected MDMs responded with a robust innate defense with biphasic p-IRF3(S386) and IRF7 induction/phosphorylation culminating in a sustained type-I IFN response, resembling the PVSRIPO innate pattern in A375 cells (Fig. 2A, B, and G). The sequence of this innate signaling cascade had an earlier start in MDMs compared to A375 cells [p-IRF3(S386) peaked at or before ~12 hpi vs ~40 hpi] (Fig. 2D and G). This could be due to far higher levels of MDA5 at baseline (and after induction) in the former (Fig. 2G). Accelerated initial p-IRF3(S386) signal in PVSRIPO-infected MDMs led to substantially faster downstream peak IFN-β synthesis, IRF7 induction, and initial p-IRF7(S477) or MDA5 induction (Fig. 2A, B, and G).

Taken together, our observations show that deficient early eIF4G cleavage of PVSRIPO is associated with MDA5-TBK1-IRF3 signaling culminating in sustained IRF7 induction and IFN-α synthesis. They indicate that A375 cells accurately reflect host type-I IFN responses to PVSRIPO and wild-type EVs and, thus, are a suitable model for mechanistic investigations of innate defenses elicited by CAV21 vs PVSRIPO.

### EV eIF4G cleavage occurs immediately after vRNA entry, prior to the onset of processive viral translation

**Fig 3:**
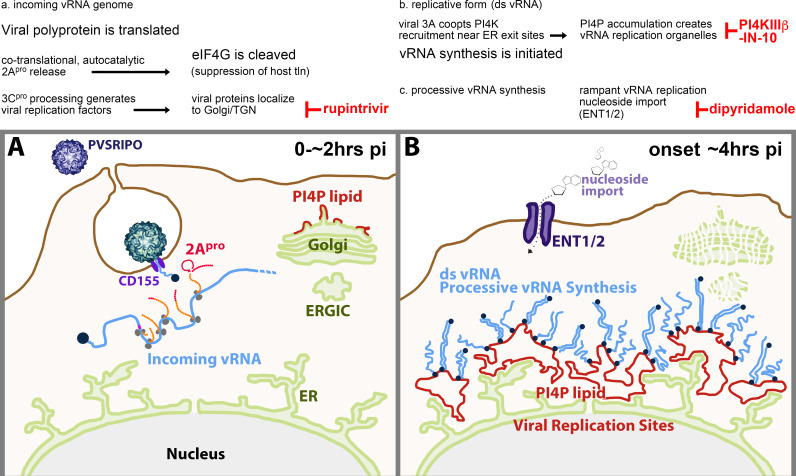
EVs employ distinct translation sites in infected host cells (adapted from reference 32). (**A**) Upon virion:CD155 binding, incoming vRNA is released into the cytoplasm from endocytic vesicles/plasma membrane invaginations. Incoming vRNA is translated upon binding of eIF4G to the viral IRES (9, 33); 2A^pro^ is released auto-catalytically from the nascent polyprotein. Rupintrivir blocks viral polyprotein processing. (**B**) PV co-opts PI4KIIIβ for generating endoplasmic reticulum (ER)-derived membrane scaffolds enriched in PI4-phosphate (PI4P) lipids, providing tethers for vRNA replication sites (32). ER-bound, PI4P-enriched viral replication sites form the platform for vRNA replication. PI4KIIIβ-IN-10 represses PI4P synthesis. Processive vRNA replication requires nucleoside import via the equilibrative nucleoside transporters 1 and 2 (ENT1/ENT2), which are inhibited by dipyridamole.

Our studies suggest immediate eIF4G cleavage as a pivotal factor in shaping innate defenses against EVs. CAV21/HRV16 efficiently thwart type-I IFN responses, while PVSRIPO’s failure to cleave eIF4G is associated with sustained IFN-α release. EV eIF4G cleavage is directed by 2A^pro^ (28), which approaches its substrate via the eIF4G binding partner eIF3 (29). Upon entry, incoming vRNA is translated and 2A^pro^ is auto-catalytically released from the nascent polyprotein by co-translational *cis*-cleavage at its N-terminus (30, 31) (Fig. 3A). PVSRIPO fails to cleave eIF4G efficiently at any stage of the infection in A375 cells, despite abundant viral proteins being generated at 6–48 hpi (Fig. 2A and B). Meanwhile, complete CAV21-mediated eIF4G cleavage occurs even before viral protein synthesis is detectable (Fig. 1A through D). This suggests that eIF4G cleavage depends on the specific circumstances of viral translation at incoming vRNA, e.g., subcellular localization and/or eIF4G/eIF3 recruitment. To test this hypothesis, we investigated eIF4G cleavage in the presence of an inhibitor of viral polyprotein processing.

### Inhibiting EV polyprotein processing does not prevent eIF4G cleavage

While 2A^pro^ accounts for proteolytic processing of EV polyproteins at two sites (31), all remaining cleavages but one are carried out by 3C^pro^. Inhibiting 3C^pro^ with the selective, irreversible inhibitor rupintrivir (34) does not interfere with entry, translation of incoming vRNA or 2A^pro^ auto-catalytic release (Fig. 3A). However, it prevents all subsequent steps in the EV life cycle leading to hijacking of phosphatidyl-inositol 4-kinase IIIβ for generating PI4-phosphate-enriched membrane structures that serve as vRNA replication platforms (Fig. 3B).

**Fig 4:**
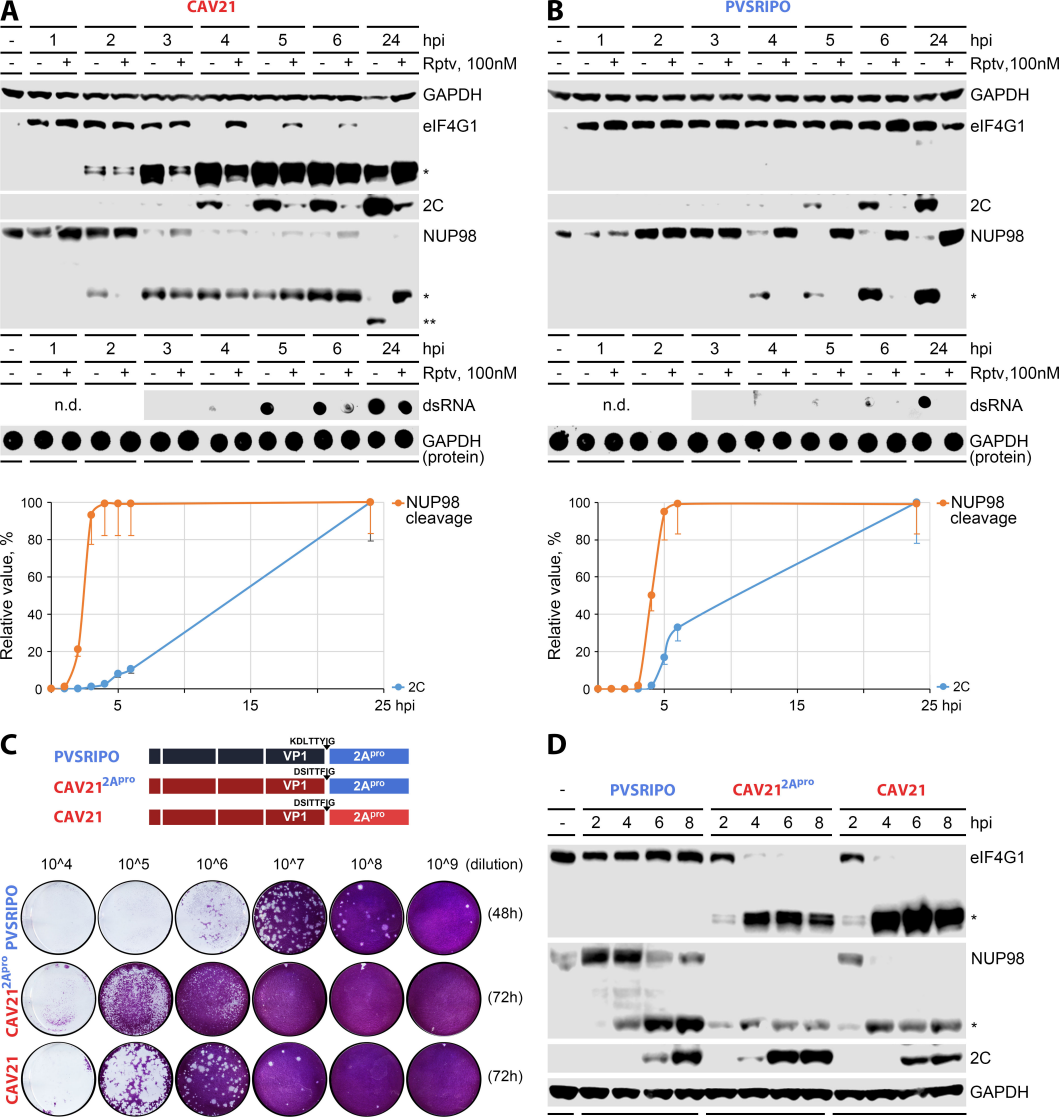
Effect of 3C^pro^ inhibition and 2A^pro^ origin on eIF4G and NUP98 cleavage (see Fig. S2 for extended data). A375 cells were infected with CAV21 (**A**) or PVSRIPO (**B**) in the absence or presence of 100 nM rupintrivir (Rptv). See Fig. S2 for dose-finding investigations. (Top panels) Cell lysates were analyzed by immunoblot for viral translation (2C) and 2A^pro^-mediated NUP98 and eIF4G cleavage (*eF4G1 and NUP98 cleavage fragments, **NUP98 fragment detected in CAV21-infected cells with overt cytotoxicity). GAPDH was used as loading control. (Middle panels) Viral dsRNA synthesis was analyzed by dot blot of the same lysates; GAPDH dot blots served as loading controls. (Bottom panels) Viral 2C expression and NUP98 cleavage were quantified in Rptv-untreated cells (% max. detected, normalized to GAPDH; means ± standard error of the mean). All assays were performed in at least three independent series; representative series are shown. (**C**) CAV21 carrying the poliovirus type 1 (Sabin) 2A^pro^ of PVSRIPO separated from VP1 by its own cleavage motif (top panel) exhibited efficient growth in HeLa cells with a very small plaque phenotype (compared to wild-type CAV21, bottom panel). (**D**) CAV21 with its cognate 2A^pro^ or the poliovirus type 1 (Sabin) 2A^pro^ yielded similar kinetics of eIF4G and NUP98 cleavage.

We used rupintrivir to investigate 2A^pro^ cleavages in CAV21 vs PVSRIPO-infected A375 cells at a dose of 100 nM (see Fig. S2 for dose titration [Fig. S2A and B] and estimates of inhibitory constants for viral translation [Fig. S2C] and dsRNA accumulation [Fig. S2D]). We analyzed viral polyprotein processing in infected A375 cells to confirm that rupintrivir (100 nM) leads to the accumulation of unprocessed P3 precursor in CAV21-infected cells, and a decrease in (final processing product) 3D^pol^ in CAV21/PVSRIPO-infected cells (Fig. S2E and F). In addition to eIF4G, we also tested the second prominent host feature targeted for immediate 2A^pro^ cleavage, the nuclear pore (35), and NUP98 (29) specifically (Fig. 4). In CAV21-infected A375 cells, rupintrivir profoundly diminished viral translation and vRNA replication, as shown by dot blot detection of the ds-vRNA replicative intermediate (Fig. 4A). However, eIF4G and NUP98 cleavages were only minimally delayed, indicating that they occur prior to complete polyprotein processing (Fig. 4A).

Rupintrivir (100 nM) abolished viral translation/RNA replication of PVSRIPO. eIF4G cleavage did not occur in untreated cells, despite abundant viral protein synthesis in the processive phase, implying the presence of 2A^pro^ (Fig. 4B). However, late NUP98 cleavage in-step with processive viral translation at 4–24 hpi was observed (Fig. 4B). This divergence may be explained by the finding that, in contrast to eIF4G, 2A^pro^ binds NUP98 directly (29), and cleavage may not depend on the specific context of eIF3-eIF4G assembly (joining 2A^pro^ with its eIF4G substrate [29]) during translation of incoming vRNA.

Thus, CAV21 eIF4G cleavage occurs during translation of incoming vRNA, prior to complete polyprotein processing and vRNA replication (<3 hpi, Fig. 4A). PVSRIPO’s deficit defining its host relationship is the inability to carry out this early eIF4G cleavage. Even during processive translation in cells with rampant type-I IFN responses (~4–24 hpi), eIF4G remains cleavage resistant (Fig. 4B). Nuclear pore degradation during processive viral translation in infected A375 cells (Fig. 4B) does not interfere with the robust antiviral type-I IFN response elicited by PVSRIPO (Fig. 2A and B). We tested whether intrinsic deficits of PVSRIPO’s 2A^pro^ mediate its eIF4G/NUP98 cleavage phenotype using a mix-and-match recombinant featuring the PVSRIPO 2A^pro^ coding sequence within a CAV21 background (Fig. 4C and D). This chimeric virus was viable with efficient growth and cytotoxicity in HeLa cells, albeit a very small plaque phenotype (Fig. 4C). PVSRIPO 2A^pro^ in a CAV21 background-mediated eIF4G/NUP98 cleavage with kinetics resembling wild-type CAV21 (Fig. 4D). Thus, PVSRIPO’s host protein cleavage phenotype is the result of early viral translation deficits mediated by the foreign HRV2 IRES and not due to performance of its cognate 2A^pro^.

### Inhibiting PI4KIIIβ or ENT1/ENT2 rescues type-I IFN responses to wild-type EV infection

**Fig 5:**
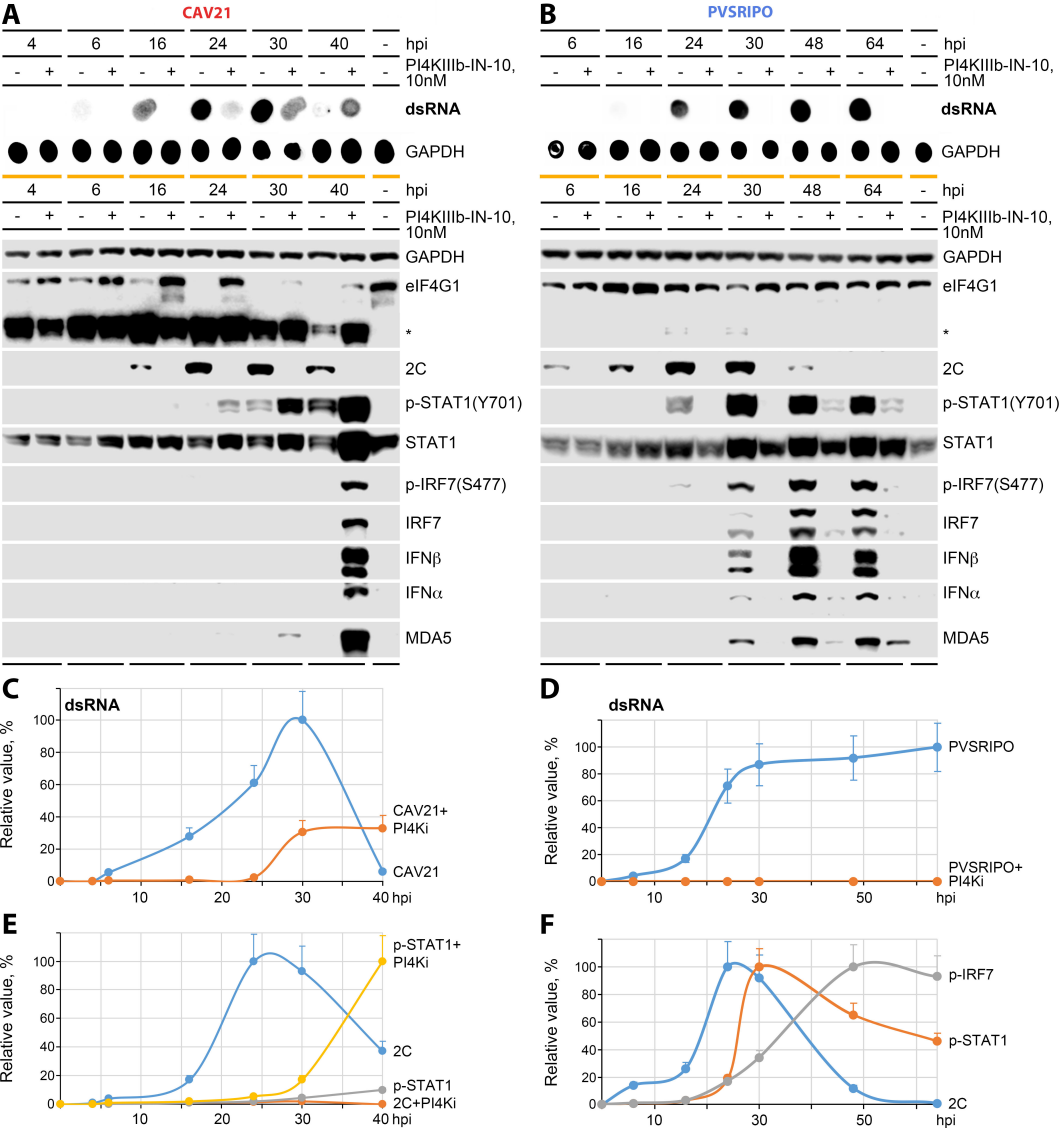
Effect of PI4KIIIβ inhibition on the innate antiviral type-I IFN response to CAV21 and PVSRIPO (see Fig. S3 for extended data). A375 cells were infected with CAV21 (A) or PVSRIPO (B) as described for Fig. 2 in the presence or absence of 10 nM PI4KIIIβ-IN-10. Cell lysates collected at the indicated hpi were analyzed by dot blot for accumulation of viral dsRNA (upper panels) or by immunoblot for viral protein synthesis (2C), eIF4G cleavage, and the innate antiviral response [p-STAT1(Y701), p-IRF7(S477), IFN-α/IFN-β, and MDA5] (bottom panels; *eIF4G1 cleavage products). All assays were performed in at least three independent series; representative results are shown. Levels of dsRNA accumulation (C, CAV21; D, PVSRIPO), levels of p-STAT1(Y701)/2C in the presence of absence of inhibitor (E, CAV21), and levels of pIRF7(S477)/p-STAT1/2C in mock-treated cells (F, PVSRIPO) were quantified and plotted in the graphs (% max. normalized to GAPDH, means ± standard error of the mean).

While deficient immediate eIF4G cleavage defines PVSRIPO’s accentuated type-I IFN imprint, this deficit does not explain the source and unusual kinetics of this response. We used the PI4KIIIβ inhibitor PI4KIIIβ-IN-10 to block the formation of PI4P-enriched replication organelles/vRNA replication (Fig. 3B) and assessed the kinetics of dsRNA accumulation, eIF4G cleavage, viral translation, innate signaling response, and host type-I IFN release in A375 cells (Fig. 5). Dose titration studies of PI4KIIIβ-IN-10 revealed higher sensitivity of dsRNA accumulation for PVSRIPO (IC_50_, ~5 nM) vs CAV21 (IC_50_, ~22 nM) (Fig. S3). We deliberately used a PI4KIIIβ-IN-10 dose with incomplete suppression of vRNA replication (10 nM, Fig. S3) to gauge the ability of CAV21 vs PVSRIPO to withstand PI4K inhibition and to induce innate host type-I IFN responses.

PI4K inhibition had a minor effect on eIF4G cleavage by CAV21, consistent with this event occurring prior to PI4P enrichment, upon translation of incoming vRNA (Fig. 5A). In contrast, CAV21 dsRNA accumulation (up to 24 hpi) and processive viral translation were profoundly suppressed (Fig. 5A, C, and E). As shown in Fig. 2, CAV21 infection of mock-treated cells led to blunted p-STAT1(Y701) without downstream IRF7 induction or type-I IFN release (Fig. 5A and E). P-STAT1(Y701) at 24–40 hpi in mock-treated cells likely does not reflect type-I IFN signaling (IFN-α/IFN-β were not detected; Fig. 5A) and may be due to cytotoxicity.

In accordance with our dose-titration assays (Fig. S3), PI4K inhibition with 10 nM PI4KIIIβ-IN-10 did not block CAV21 vRNA replication permanently. DsRNA accumulation was undetectable before 24 hpi in the presence of drug but gradually increased at 24–40 hpi (Fig. 5A and C). At 40 hpi in the presence of 10 nM PI4KIIIβ-IN-10, a dose that blocked processive viral translation (at all times) and vRNA replication (up to 24 hpi), IRF7 induction, and type-I IFN production occurred (Fig. 5A). Thus, profound suppression of vRNA replication protects cells from CAV21 cytopathogenicity, enabling delayed dsRNA accumulation that triggers innate antiviral type-I IFN defenses (akin to PVSRIPO). This response may be possible because of the presence of intact eIF4G in CAV21-infected, PI4KIIIβ-IN-10-treated cells, due to either remnant protein or the induction of eIF4G biosynthesis in infected cells in the presence of PI4K inhibition (Fig. 5A). Indeed, IRES activity has been shown to drive expression of eIF4G1 isoforms generated by a series of in-frame initiation codons (36). Thus, in CAV21-infected cells protected from viral processive translation/cytopathogenicity by PI4KIIIβ-IN-10, active cap-independent translation of eIF4G1 may enable type-I IFN responses.

In contrast to CAV21, PI4K inhibition abolished all PVSRIPO activities other than a weak, late type-I IFN response (Fig. 5B and D). Due to deficient translation of incoming vRNA in A375 cells, PVSRIPO cannot overcome PI4K inhibition in the manner observed with CAV21 at the same dose of PI4KIIIβ-IN-10. Thus, no dsRNA was detected in the presence of PI4K inhibitor (1–64 hpi) (Fig. 5D), and the innate STAT1-IRF7 antiviral response was almost completely blocked (Fig. 5B and D). In untreated cells, the PVSRIPO-induced sustained p-IRF7(S477)/IFN-α/IFN-β response (Fig. 5B and F) correlated with ongoing dsRNA accumulation (Fig. 5D). Thus, type-I IFN responses to CAV21 (induced by PI4K inhibition) and to PVSRIPO are enabled by preservation of intact eIF4G and the accumulation of viral dsRNA; they do not correlate with the kinetics of viral translation.

PI4KIIIβ-IN-10 treatment of HeLa cells caused delayed PVSRIPO dsRNA accumulation and IRF3-STAT1 phosphorylation/activation (Fig. S4A) in a similar manner as observed for CAV21 in A375 cells (Fig. 5A). Thus, PI4K inhibition converted PVSRIPO’s cytopathogenic profile in HeLa cells (Fig. 1A) toward the accentuated MDA5 signaling phenotype in A375 cells (Fig. S4A). Type-I IFN responses to CAV21 and PVSRIPO mirrored the pace of dsRNA accrual in all models/conditions, consistent with evidence that the EV replicative intermediate is the pattern sensed by MDA5 (1). Conversely, inhibiting TBK1 with Bx795 (19) in A375 cells converted the accentuated MDA5 signaling phenotype of PVSRIPO into a cytopathogenic program with enhanced early viral translation and efficient eIF4G cleavage (Fig. S4B).

**Fig 6:**
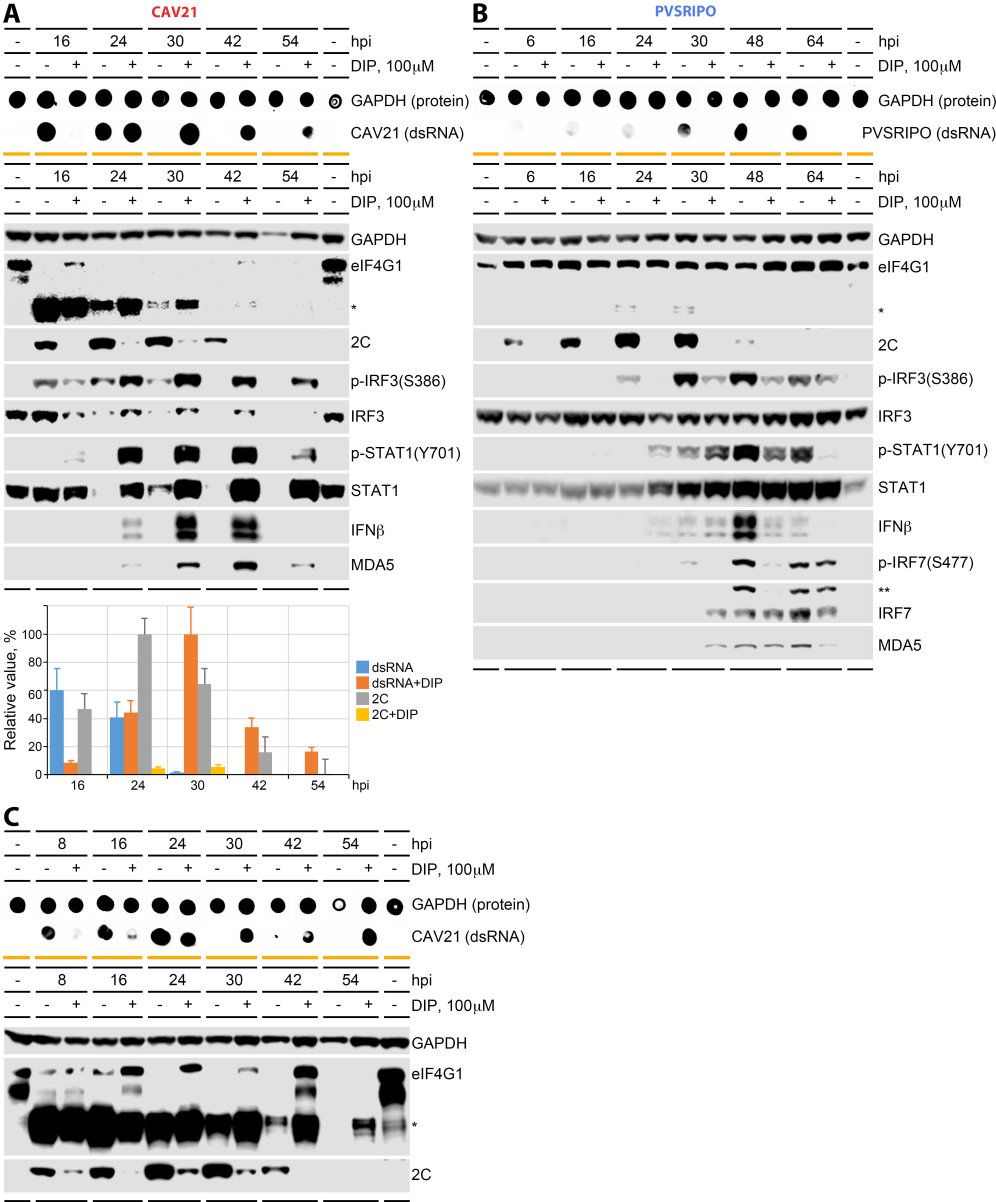
The effect of inhibiting vRNA replication with dipyrimadole (DIP) on innate type-I IFN responses to CAV21 and PVSRIPO. Time course of A375 infection with CAV21 (A) or PVSRIPO (B) performed in a manner similar to that in Fig. 5 in the absence (dimethyl sulfoxide[DMSO]) or presence of 100 μM DIP. Cell lysates were analyzed by dot blot for vRNA replication (dsRNA, top panels) and by immunoblot for eIF4G cleavage, viral translation (2C), and innate type-I IFN response signatures (middle panels; *eIF4G1 cleavage products, **p-IRF7 with decreased electrophoretic mobility detected with IRF7 antibody). Assays were performed in at least three independent series; representative results are shown. Accumulation of dsRNA and 2C protein in CAV21-infected cells was quantified from three independent experiments and plotted in the bottom graph (% max. normalized to GAPDH, means ± standard error of the mean). (C) A repeat series of the assay shown in panel A with high detection sensitivity was conducted to rigorously document eIF4G1 status in CAV21-infected cells in the presence of DIP.

We extended our findings with inhibition of 3D^pol^-direted vRNA synthesis by dipyridamole (DIP) treatment of A375 cells. Broad antiviral activity of DIP, due to inhibition of nucleoside import by ENT1/ENT2 (Fig. 3B) (37), was shown in 1977 (38). DIP dose titration assays informed our decision to use a concentration of 100 µM with the intent to prevent cytotoxicity and produce host innate type-I IFN responses to CAV21 at 24 and 48 hpi (Fig. S5A). In CAV21-infected A375 cells, DIP severely curtailed processive viral translation (at any time) and dsRNA accumulation (up to 16 hpi, Fig. 6A and C). However, dsRNA accumulation in the presence of 100 µM DIP recovered by 24 hpi (Fig. 6A and C). At 30 hpi, it increased further, followed by a gradual decrease (Fig. 6A). Late dsRNA accrual in DIP-treated cells occurred in step with a robust type-I IFN response (Fig. 6A). As observed with PI4K inhibition, this may be due to partially preserved host cell integrity (due to inhibition of processive viral translation) and retention of intact eIF4G (Fig. 6A).

To examine eIF4G status specifically, the assay shown in Fig. 6A was repeated with measures to achieve very high eIF4G detection sensitivity (Fig. 6C). This revealed the absence of intact eIF4G in mock-treated CAV21-infected cells (after 16 hpi), while intact eIF4G was preserved, or even induced (see 42 hpi), in DIP-treated samples (Fig. 6C). These observations re-affirm our hypotheses that early EV eIF4G cleavage abolishes the antiviral type-1 IFN response and that preservation of eIF4G integrity in PVSRIPO-infected cells enables complex, MDA5-orchestrated TBK1-IRF3-IRF7 signaling and sustained IFN-α release.

As indicated in the DIP dose-finding studies (Fig. S5), DIP (100 µM) treatment of A375 cells abolished PVSRIPO translation and vRNA replication and, accordingly, greatly diminished IFN-β synthesis (Fig. 6B).

Our investigations revealed that PVSRIPO-mediated IRF3-IRF7 activation and sustained type-I IFN release are the results of an extreme level of attenuation far beyond the naturally non-cytopathogenic (in human airway epithelium) HRV16. The failure of the virus to execute immediate eIF4G cleavage, mediating loss of cytopathogenicity in relevant PV target host cells, produces non-cytotoxic dsRNA accumulation that triggers unopposed MDA5 engagement.

## DISCUSSION

EVs use a unique, highly effective strategy of thwarting innate antiviral type-I IFNs through drastic host interference via 2A^pro^-directed host protein cleavage. Our investigations indicate that immediate eIF4G cleavage is necessary and sufficient to abrogate type-I IFN release in EV-infected cells. Inhibiting polyprotein processing with rupintrivir and interfering with vRNA replication via PI4K or ENT1/ENT2 inhibition profoundly diminished processive viral translation/dsRNA accumulation but only modestly affected eIF4G/NUP98 cleavage in CAV21-infected cells. This demonstrates that the 2A^pro^-directed host protein cleavages occur upon initial translation of incoming vRNA, prior to complete polyprotein processing and the onset of vRNA replication. A persistent conundrum in the field has been the stoichiometry of the 2A^pro^-substrate relation, where near-complete cleavage of the entire pool of eIF4G present in the cell is executed by a limited 2A^pro^ supply early in the infectious cycle [reviewed in reference (39)]. The fact that abundant PVSRIPO translation during the processive phase of viral protein synthesis (~4 to 24 hpi) fails to execute eIF4G—but not NUP98—cleavage, as well as the finding of 2A^pro^:eIF3 binding as a possible requirement for cleavage (29), hints at possible explanations. We speculate that the specific conditions for initial translation of incoming vRNA, e.g., the context provided by subcellular location or the manner of eIF4G:eIF3 engagement during initial viral translation, are necessary for immediate 2A^pro^-directed eIF4G cleavage to occur.

Our investigations point to deficient immediate viral translation at incoming vRNA as the root cause for PVSRIPO’s inability to cleave eIF4G. This phenotype was linked to stem-loop domains (SLDs) 5 and 6 in the HRV2 IRES (40). We showed previously that SLDs 5 and 6 of EV IRESs, the footprint for binding to the eIF4G translation initiation scaffold (9, 19, 40), bind the dsRNA-binding protein kinase (PKR) (19). Upon PKR binding to the IRES, PKR dimerization and auto-phosphorylation ensue in a manner that interferes with translation initiation at the IRES, possibly via steric hindrance of eIF4G binding to stem loop domains 5 and 6 (19). We suspect that a relative deficiency in recruiting eIF4G early exposes PVSRIPO RNA genomes to PKR sensing, thus delaying immediate translation of incoming vRNA and protecting eIF4G from 2A^pro^ cleavage.

Comparing CAV21, HRV16, and PVSRIPO innate antiviral immunity in the same host (A375 cells) side-by-side revealed the consistency of the EV eIF4G cleavage paradigm and PVSRIPO’s profound attenuation. Despite ~10-h delayed eIF4G cleavage (relative to CAV21) and efficient IRF3(S386) phosphorylation—roughly at par with PVSRIPO—a type-I IFN response to HRV16 was almost completely absent. This suggests that eIF4G cleavage, at a level and with timing commensurate with preventing host type-I IFN release, defines the *Enterovirus* genus.

Comparing CAV21 with PVSRIPO in the presence of PI4K or ENT1/ENT2 inhibition also revealed an extreme level of attenuation for the latter. The accentuated MDA5-IRF3-IRF7 signature of PVSRIPO in A375 cells was achieved with CAV21 only when host cell integrity was preserved, processive viral translation nearly abrogated, and vRNA replication markedly impeded by PI4KIIIβ or ENT1/ENT2 inhibition. As in the case of PVSRIPO, innate type-I IFN responses to (PI4K or ENT1/ENT2 inhibition-impeded) CAV21 correlated with the rate of viral dsRNA accumulation.

PVSRIPO exhibits cytopathogenic properties in many high-passage, tissue culture-adapted tumor models, such as HeLa cells. Elucidation of mitogenic signaling cascades converging on translation initiation machinery revealed that uncontrolled PKC-Raf-ERK1/2-MNK1/2 signaling—a hallmark of the malignant state (41)—spurs early viral IRES-mediated translation in PVSRIPO-infected cells and, thus, favors eIF4G cleavage (42–49). It is unclear if the prevailing conditions for protein synthesis control in high-passage cancer cell lines (eg., HeLa cells) are representative of neoplastic cells in their native habitat or if they are skewed by serial passage adaptations. PVSRIPO treatment of glioblastoma patient *ex vivo* tumor slices—an undissociated, non-passaged authentic representation of the heterogeneous mix of cells present in the tumor—revealed the absence of cytotoxicity in all tumor compartments, including neoplastic cells (12).

Given its fundamentally altered host-innate relations, it is highly improbable that PVSRIPO could establish a gastrointestinal replication reservoir—let alone transmission—consistent with the lack of virus shedding in clinical trials of PVSRIPO in recurrent glioblastoma (50) and melanoma (51, 52). Without the eIF4G cleavage paradigm—the defining characteristic of the *Enterovirus* genus shaping EV-host innate relations—the genetically engineered, non-cytopathogenic PVSRIPO may be considered axiomatically distinct from its relatives.

## MATERIALS AND METHODS

### Cell lines and viruses

HeLa R19, A375, Hs683, and M059J cells were sourced, grown, infected with PVSRIPO, CAV21 (Kuykendall), chimeric CAV21 containing the 2A^pro^ coding sequence of PVSRIPO (see below) or HRV16 and treated with indicated inhibitors using methods described earlier (19). Viruses were titrated by plaque assay or TCID_50_ (when comparing viruses with very different plaque size). For inhibitor studies, cultures in 60-mm dishes were subjected to an “attachment” step of 30 min at 37°C after virus suspension (2 mL, MOI of 10) was added. After this step, the cultures were rinsed with growth medium and new medium containing the inhibitor at the desired concentration was added. In the “delayed inhibition” experiment, rupintrivir was added to established infections at the indicated times before cell lysis (see Fig. S2). Infections with HRV16 were carried out at 33°C. Chimeric CAV21 containing the 2A^pro^ coding sequence of PVSRIPO was engineered as follows. Two overlapping PCR fragments generated with primer pairs: (i) CAV21^P1^/PV^2A^ linkage 5′-tcctcgagctgtattatacaggggagagggagtggacatgatatccagtgcaattctacctctgaccaaggtagactcaattaccacatttggattcggacacc-3′/5′-tgatgccctgttccatggcttcttcttcg-3′ and (ii): PV^2A^/CAV21^2B^ linkage 5′-atggaacagggcatcacaagctacatc-3′/5′-gcaaggcctttccacaaacgag-3′ were fused in second-round PCR with flanking primers 5′-tcctcgagctgtattatacagg-3′ and 5′-gcaaggcctttccacaaacgag-3′. The resulting fragment was digested with *Xho*I and *Stu*I (restriction sites in the primer sequences are underlined) and inserted into the corresponding sites of CAV21 cDNA. Chimeric virus was recovered after transfection of HeLa cells with *in vitro* transcribed vRNA. Poly(I:C) transfections were performed with high molecular weight poly(I:C)/LyoVec (InvivoGen) (12). MDMs were differentiated from “Leukopak” peripheral blood mononuclear cells (PBMCs, Stemcell Tech). After thawing, PBMCs were washed (10-mL AIM-V [Invitrogen]), incubated (15 min) in AIM-V containing 10-µg/mL DNAse I (Roche), pelleted and plated (1 × 10^6^ cells per well in six-well plates) in Dulbecco’s modified Eagle medium (DMEM; Gibco) containing 10% FBS with macrophage colony-stimulating factor (50 µg/mL, Stemcell Technologies). Cells were cultured for 6 days prior to infection.

### Inhibitors and antibodies

Rupintrivir (#PZ0315), DIP (#D9766), puromycin dihydrochloride (#P8833) (all Sigma), PI4KIIIβ-IN-10 (MedChemExpress, #HY-100198), and Bx795/912 (SelleckChem, #S1274/S1275) were dissolved in DMSO and used at the indicated concentrations. Mock treatment consisted of the corresponding concentration of DMSO in growth medium. Detailed dose titration experiments were carried out for rupintrivir (Fig. S2), PI4KIIIβ-IN-10 (Fig. S3), and DIP (Fig. S5) in A375 cells to determine the concentration suitable for use in subsequent assays. Dose titration for Bx795 in A375 cells was reported previously (19). Puromycylation assays were performed as described in Fig. S1 (53). Primary antibodies used were against eIF4G1 (#2469), GAPDH (#2118), IRF3 (#119040), p-IRF3(S386) (#37829), STAT1 (#9172), p-STAT1(Y701) (#9167), IRF7 (#49200), p-IRF7(S477) (#12390), CD54 (#67836), CD155 (#13544), IFN-β (#736710), dsRNA (K1, #28764), MDA5 (#5321), IFIT1 (#14769), and NUP98 (#2598) (all Cell Signaling Tech); and puromycin (#MABE343) (Millipore); IFN-α (#18013-1-AP), and YTHDF3 (#25537–1-AP) (both Proteintech). PV 2C and 3D antibodies were gifts from E. Wimmer, Stony Brook University, NY, and from C. Cameron, University of North Carolina at Chapel Hill, respectively.

### Immunoblot analyses

Immunoblots were carried out essentially as reported before (46, 47). Briefly, cells were lysed with polysome lysis buffer (10 mM HEPES, pH 7.4, 100 mM KCl, 5 mM MgCl_2_, 0.5% Igepal CA-630 [NP-40, Sigma], supplemented with Halt protease/phosphatase inhibitor [ThermoFisher]) followed by o/n incubation at −80°C. Lysates were centrifuged 10 min at 14,000 *× g*, and supernatants were loaded directly on nitrocellulose for dot-blot analysis or boiled with lithium dodecyl sulfate (LDS) sample buffer (ThermoFisher) for Western blots. Immunoblots were developed with SuperSignal West Pico-Plus, -Femto, or -Atto chemiluminescent substrates (ThermoFisher), depending on protein abundance/antibody sensitivity; RNA or protein bands were quantified using the Li-COR Odyssey FC imaging system and Image Studio software as described earlier (19). Briefly, protein levels (relative values) plotted on all graphs were calculated as follows: maximum detected value was set at 100%, each value was normalized to the GAPDH loading control at the corresponding time point. Calculations were performed from three independent experiments; means ± standard error of the mean are shown on the graphs.

## SUPPLEMENTAL MATERIAL

Fig. S1 mbio.01915-23-s0001.tif Quantitative assessment of active protein synthesis (puromycin incorporation) in CAV21- vs. PVSRIPO-infected A375 cells.

Fig. S2 mbio.01915-23-s0002.tif Dose-titration studies for rupintrivir.

Fig. S3 mbio.01915-23-s0003.tif Dose-finding studies for PI4KIIIb-IN-10.

Fig. S4 mbio.01915-23-s0004.tif PI4K inhibition triggers type-I IFN responses to PVSRIPO in HeLa cells; TBK1 inhibition enhances PVSRIPO cytopathogenicity in A375 cells.

Fig. S5 mbio.01915-23-s0005.tif Dose-titration studies for dipyridamole (DIP).

Supplemental legends mbio.01915-23-s0006.docx Legends for Fig. S1 to S5.

ASM does not own the copyrights to Supplemental Material that may be linked to, or accessed through, an article. The authors have granted ASM a non-exclusive, world-wide license to publish the Supplemental Material files. Please contact the corresponding author directly for reuse.
