# Supplementary figures and images for: Early enterovirus translation deficits extend viral RNA replication and elicit sustained MDA5-directed innate signaling
Source: mBio. 2023 Nov 14;14(6):e01915-23. doi: 10.1128/mbio.01915-23 (PMC10746184; doi:10.1128/mbio.01915-23)

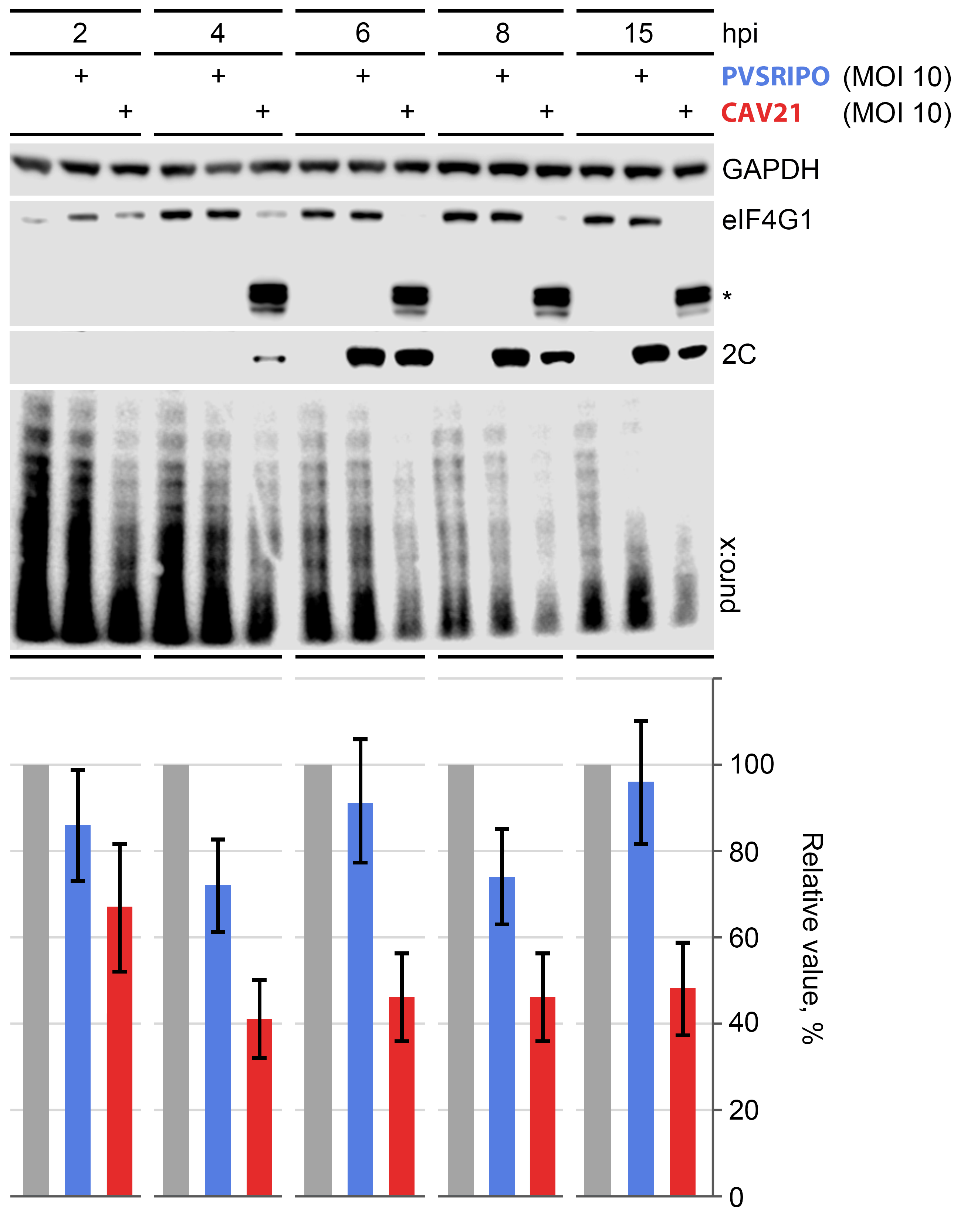

Supplement: Fig. S1 — Quantitative assessment of active protein synthesis (puromycin incorporation) in CAV21- vs. PVSRIPO-infected A375 cells. [file mbio.01915-23-s0001.tif]

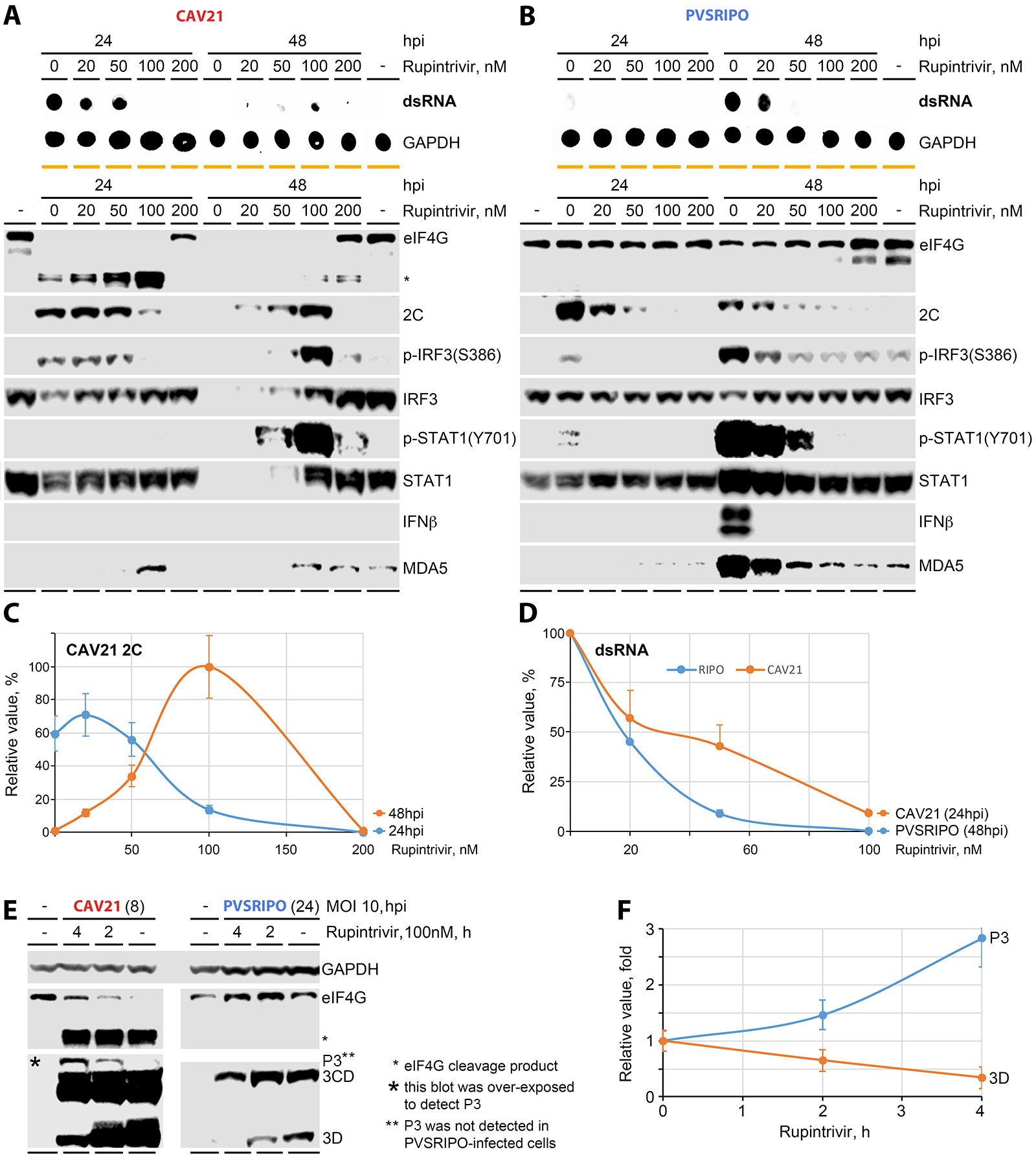

Supplement: Fig. S2 — Dose-titration studies for rupintrivir. [file mbio.01915-23-s0002.tif]

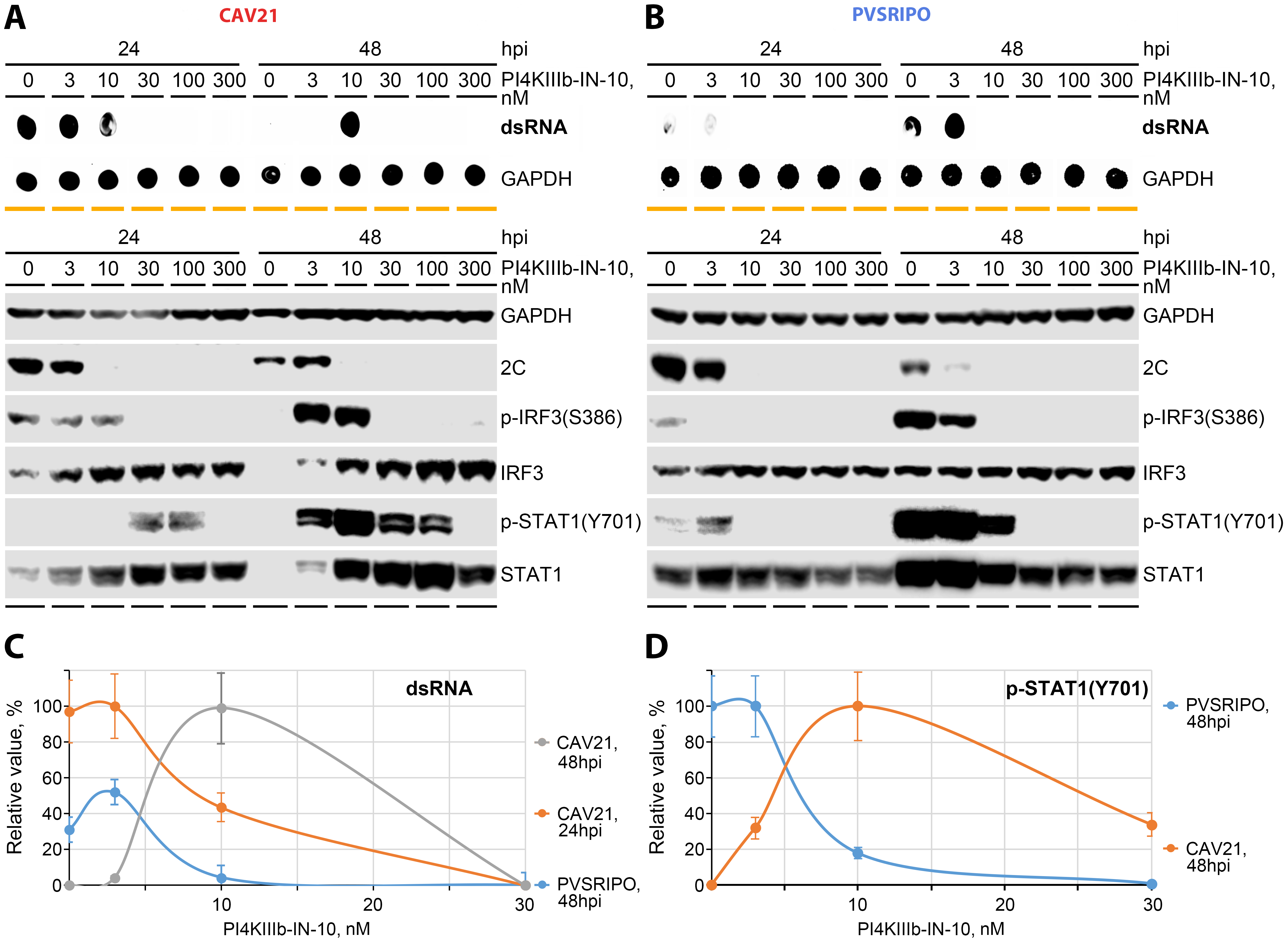

Supplement: Fig. S3 — Dose-finding studies for PI4KIIIb-IN-10. [file mbio.01915-23-s0003.tif]

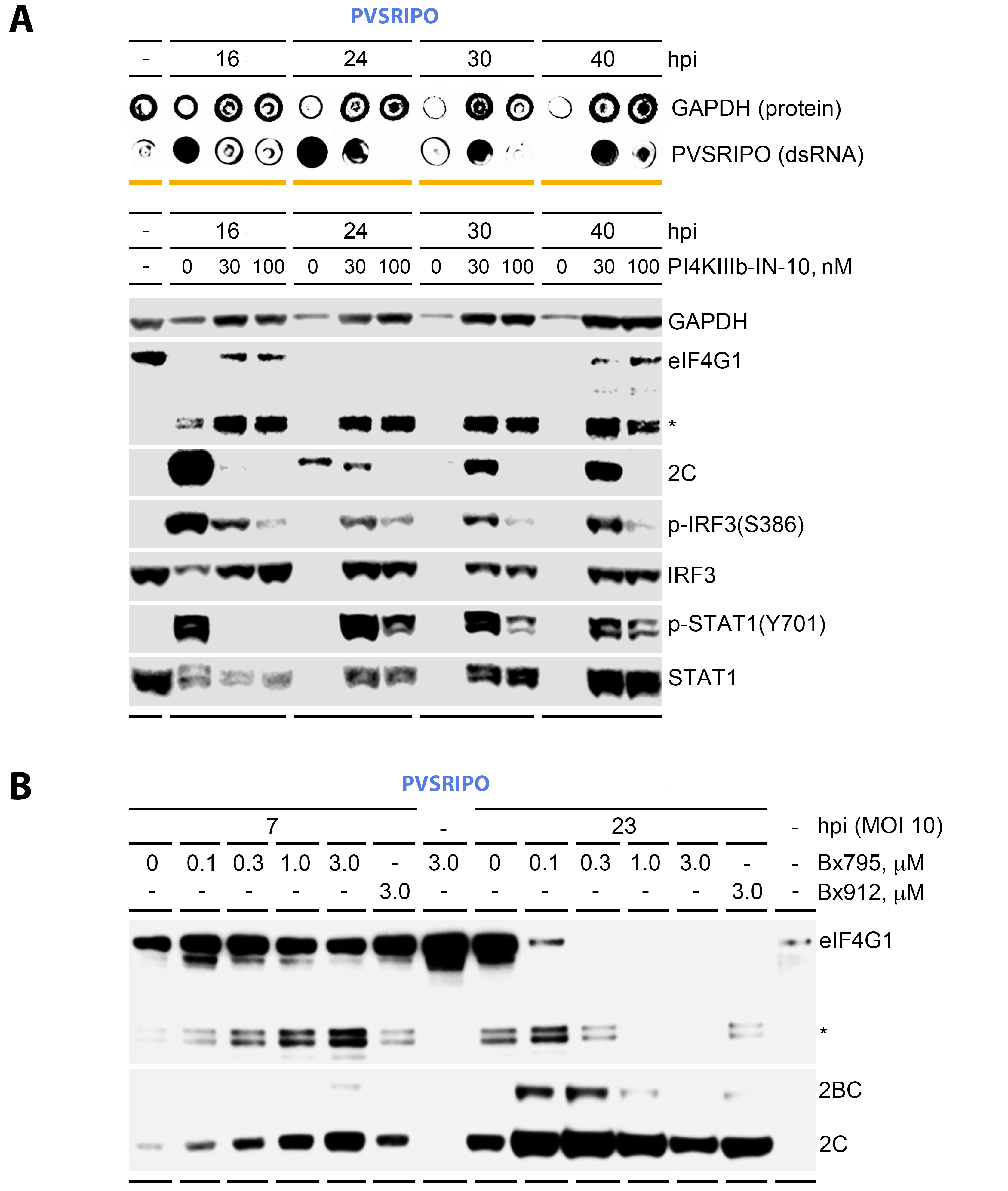

Supplement: Fig. S4 — PI4K inhibition triggers type-I IFN responses to PVSRIPO in HeLa cells; TBK1 inhibition enhances PVSRIPO cytopathogenicity in A375 cells. [file mbio.01915-23-s0004.tif]

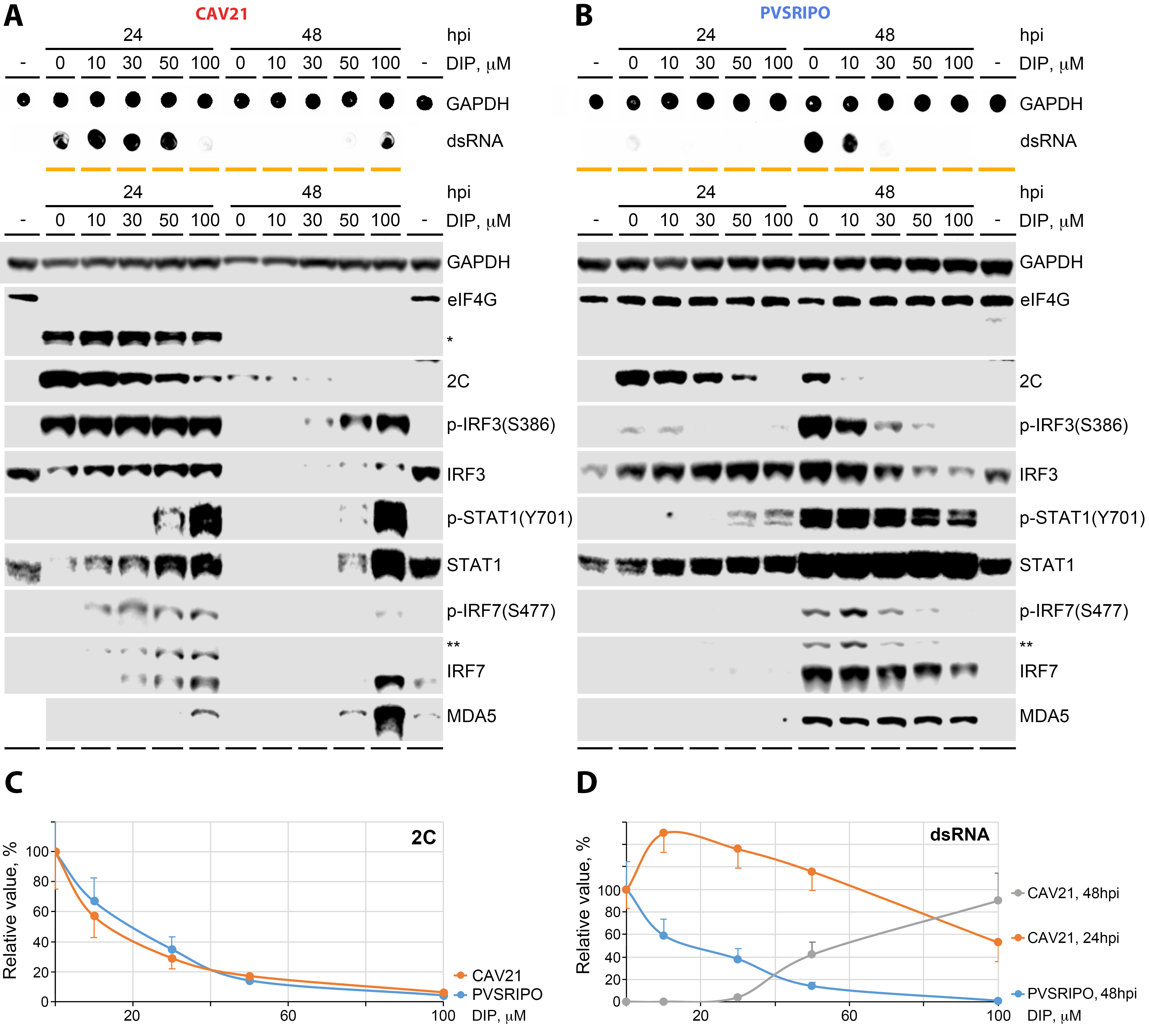

Supplement: Fig. S5 — Dose-titration studies for dipyridamole (DIP). [file mbio.01915-23-s0005.tif]
