# Supplementary material for: Early enterovirus translation deficits extend viral RNA replication and elicit sustained MDA5-directed innate signaling
Source: mBio. 2023 Nov 14;14(6):e01915-23. doi: 10.1128/mbio.01915-23 (PMC10746184; doi:10.1128/mbio.01915-23)
Supplement: Supplemental legends — Legends for Fig. S1 to S5. [file mbio.01915-23-s0006.docx]

**SUPPLEMENTAL MATERIAL**

**Early Enterovirus Translation Deficits Extend Viral RNA Replication and Elicit Sustained MDA5-Directed**

**Innate Signaling**

Dobrikov et al., 2023

**
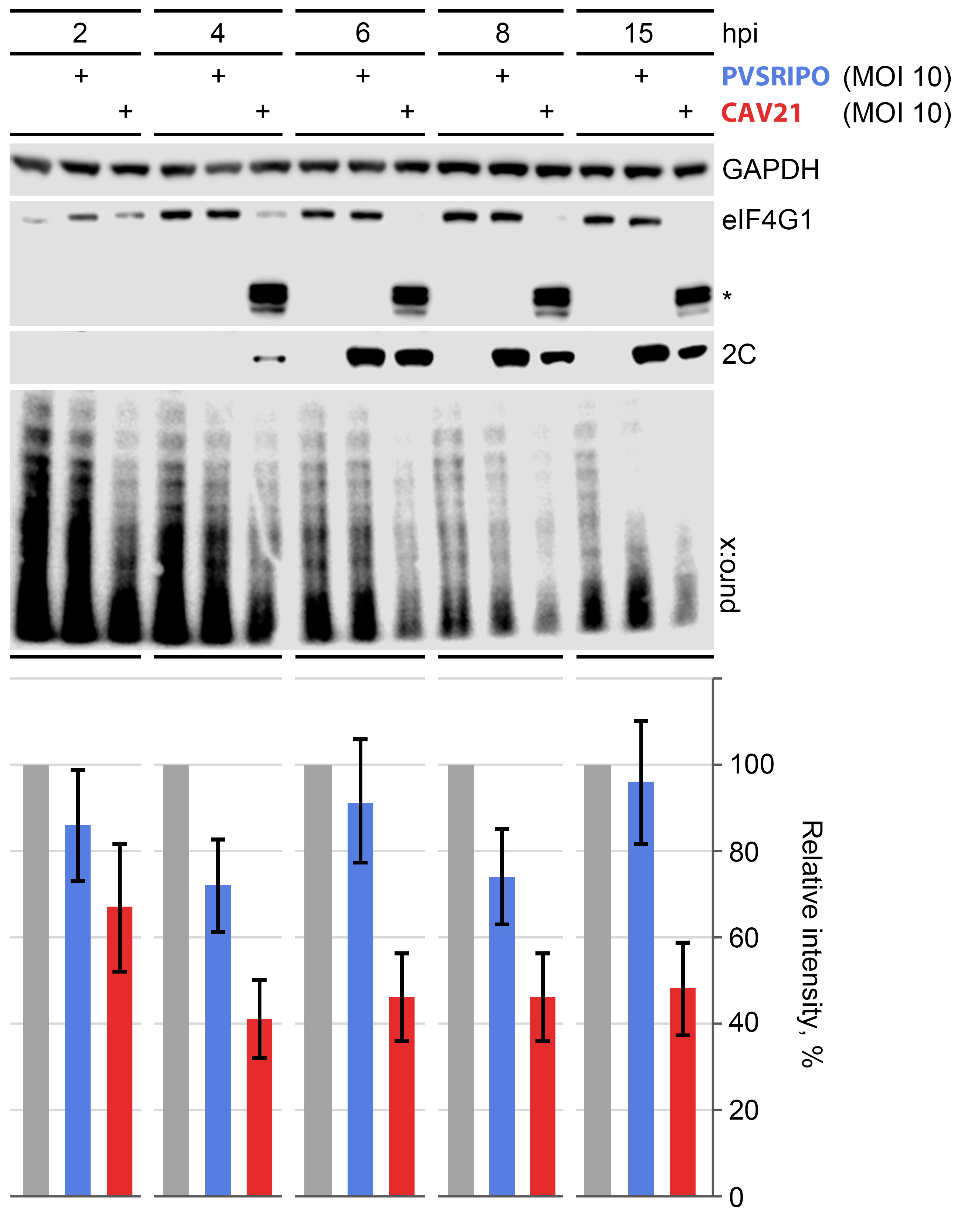
**

**Fig. S1 (related to Figure 1B). Quantitative assessment of active protein synthesis (puromycin incorporation) in CAV21- vs. PVSRIPO-infected A375 cells.** Puromycin (10 μM) was added to cells 8 min before lysis at the indicated intervals post PVSRIPO/CAV21 infection. Cells were collected at the times shown and processed for immunoblot assessing loading (GAPDH), eIF4G cleavage, viral translation (2C) and global protein synthesis activity (puromycylated polypeptides; puro:x). Puro:x proteins were quantified (%max. detected at each time point, normalized to GAPDH; means ^+^/_-_SEM). The assay was conducted in at least three independent series; representative results are shown.

**
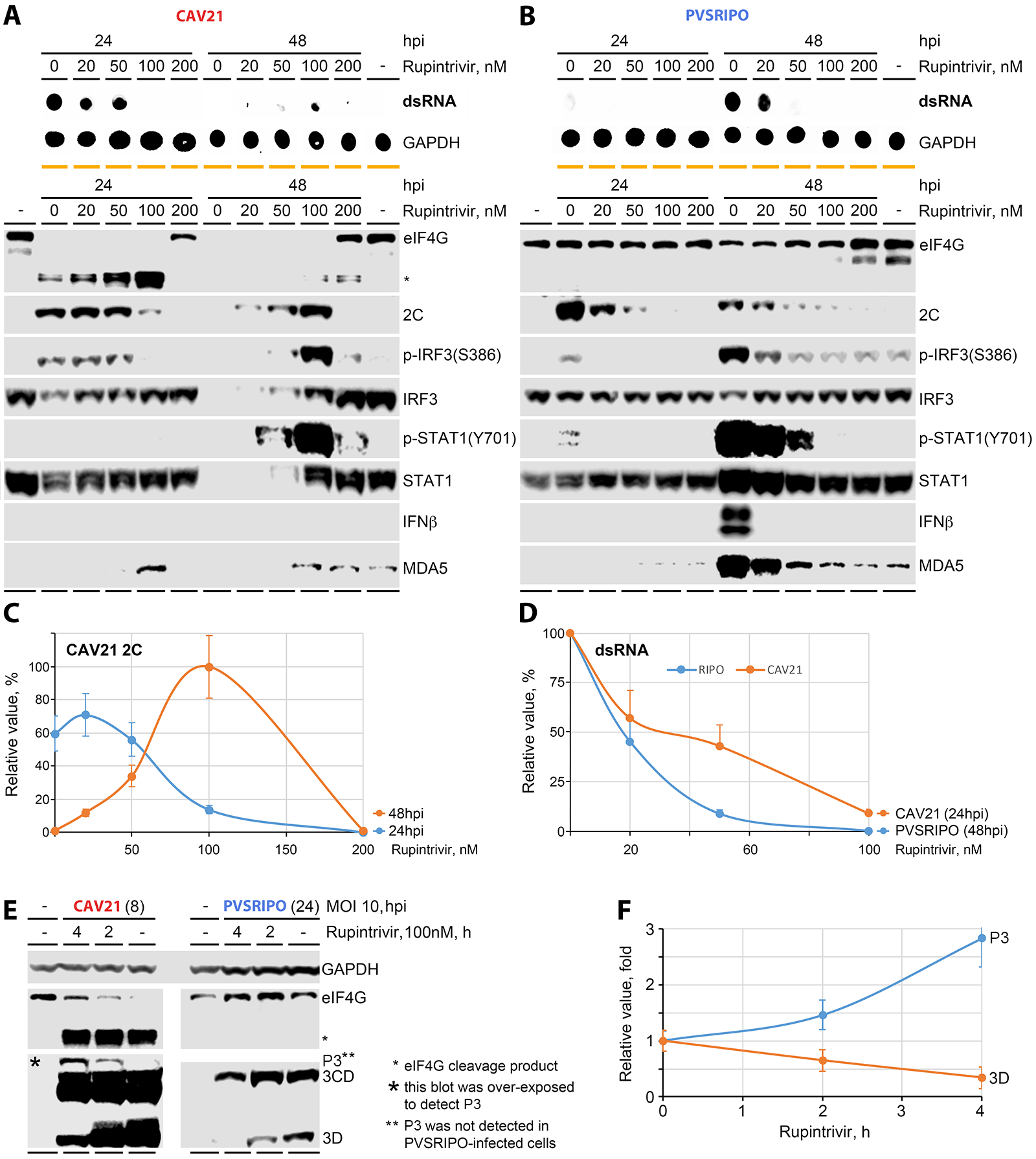
**

**Fig. S2 (related to Figure 4). Dose-titration studies of rupintrivir.** A375 cells were infected with CAV21 (MOI 10; **A**) or PVSRIPO (MOI 10; **B**) in the presence of increasing concentrations of rupintrivir (added at 0hpi). Viral dsRNA accumulation was assessed by dot blot (top panels); viral translation (2C) and innate antiviral responses were tested by immunoblot (lower panels). Quantification of viral 2C in CAV21-infected cells (**C**) and the accumulation of dsRNA in PVSRIPO/CAV21 infected cells (**D**) (%max. detected, normalized to the GAPDH loading control; means ^+^/_-_SEM). Our tests yielded IC_50_ estimates for inhibition of dsRNA accumulation of ~30 nM (CAV21, 24hpi) and ~18 nM (PVSRIPO, 48hpi) (**D**). For further tests with rupintrivir inhibition, a concentration of 100 nM was used for both viruses (see main text). The analyses were performed in triplicate and representative results are shown. (**E**) Rupintrivir inhibits viral P3 precursor processing by 3C^pro^. A375 cells were infected with CAV21 (8h) or PVSRIPO (24h) and rupintrivir was added to the infected cells for the indicated intervals before lysis. Lysates were analyzed by immunoblot for eIF4G cleavage and for 3C^pro^-mediated processing of the P3 precursor (yielding 3CD and 3D). Representative results from 3 experimental series are shown. (**F**) CAV21-mediated expression and processing of P3 and 3D were quantified in relation to rupintrivir exposure [fold difference (relative protein level without rupintrivir treatment set as 1), normalized to GAPDH; means ^+^/_-_SEM].


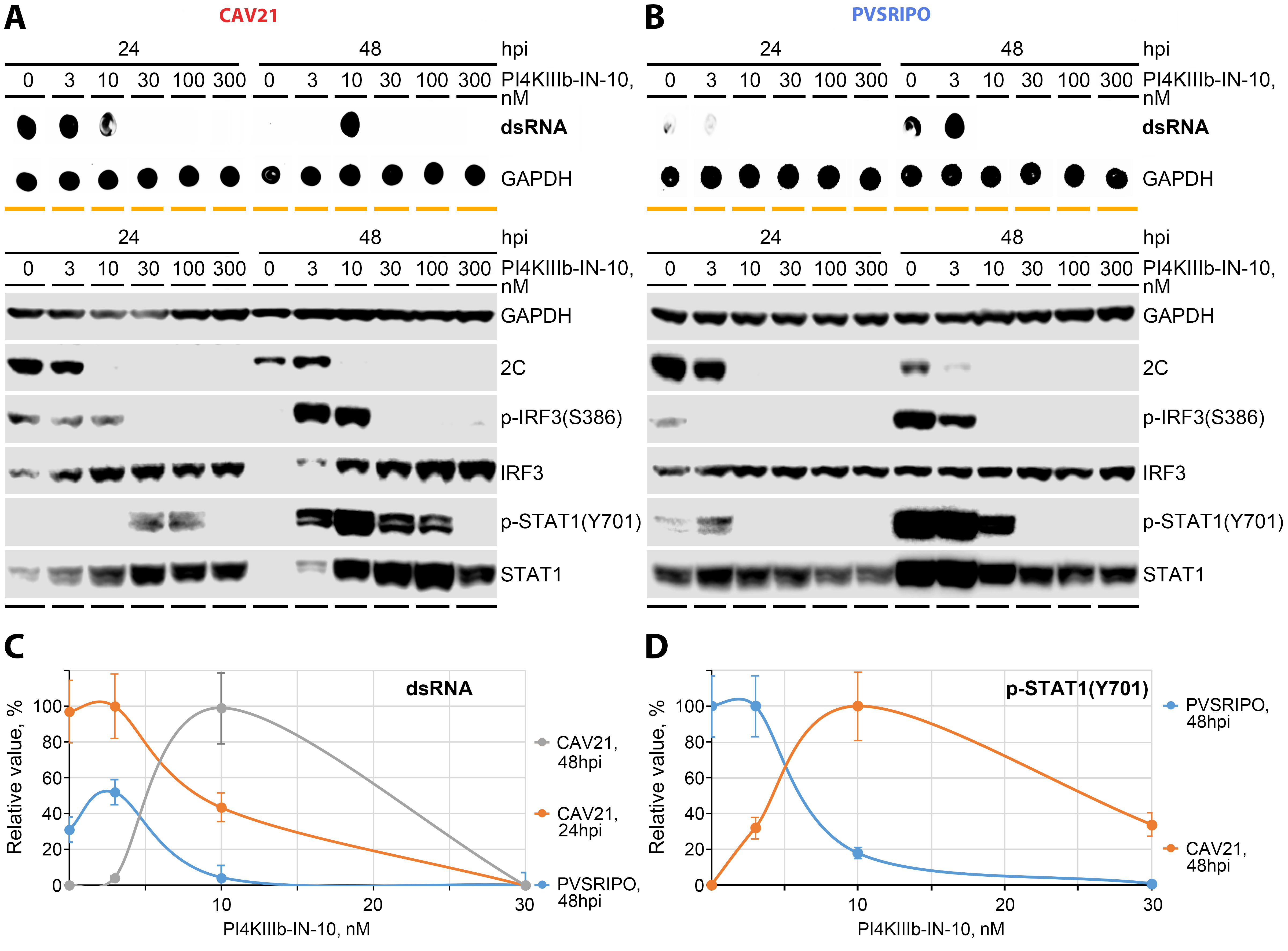


**Fig. S3 (related to Figure 5). Dose-finding studies for PI4KIIIβ-IN-10.** A375 cells were infected with CAV21 (**A**) or PVSRIPO (**B**) (MOI of 10) and treated with mock (0) or with PI4KIIIβ-IN-10 (3-300 nM) at the time of virus addition. Cells were lysed at the indicated intervals and cell lysates were processed for immunoblot of viral translation (2C) and the innate antiviral response [p-IRF3(S386)/IRF3, p-STAT1(Y701)/STAT1]. Quantification of the accumulation of dsRNA (**C**) and of p-STAT1(Y701) (**D**) (%max. detected, normalized to GAPDH; means ^+^/_-_SEM). The assays were performed in at least three independent series; representative results are shown.

**
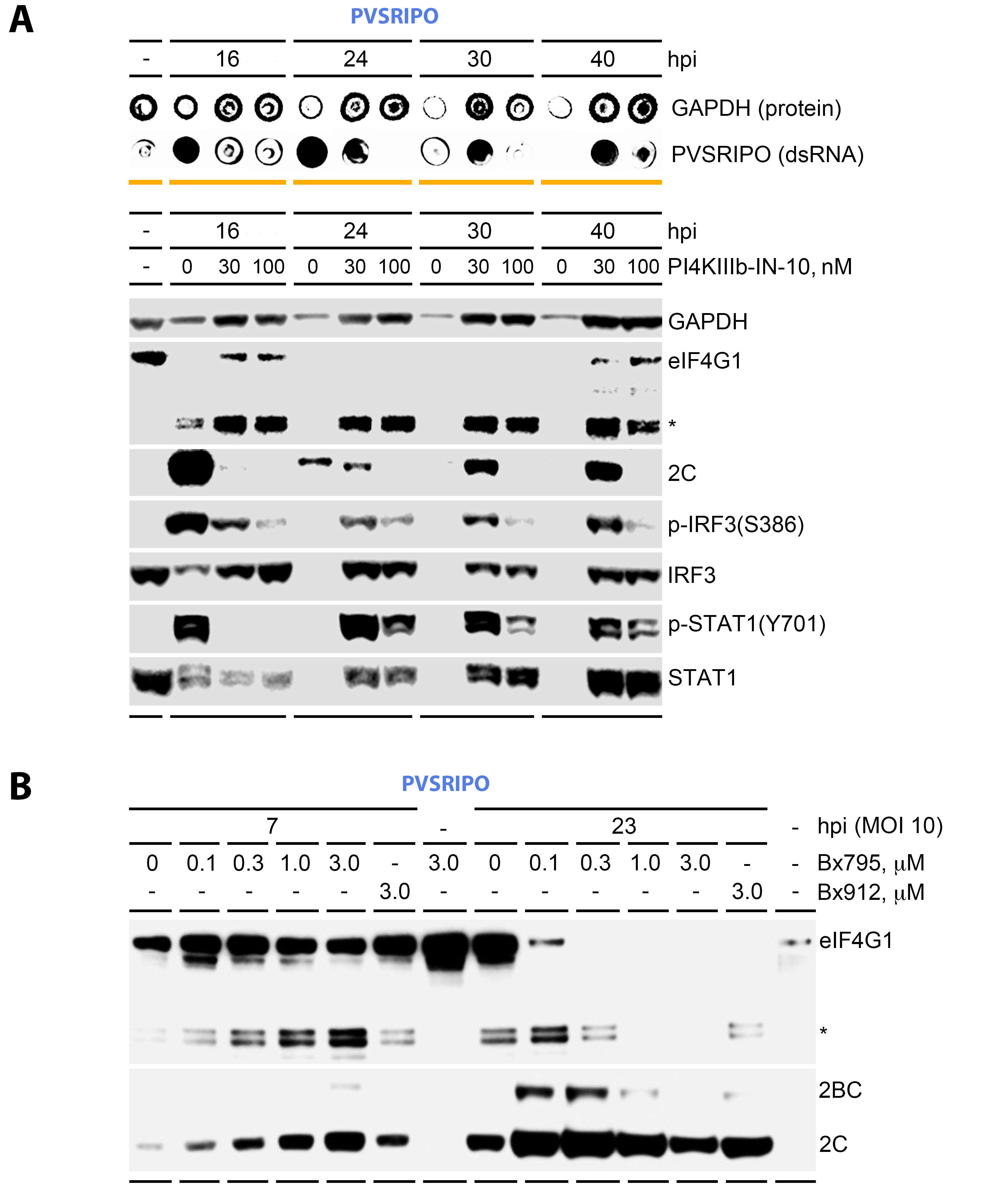
**

**Fig. S4 (related to Figures 1A, 5). PI4K inhibition triggers type-I IFN responses to PVSRIPO in HeLa cells; TBK1 inhibition enhances PVSRIPO cytopathogenicity in A375 cells.** (**A**) HeLa cells were infected with PVSRIPO (MOI 10) in the absence (DMSO) or presence of 30 nM or 100 nM PI4K inhibitor. Samples were analyzed by dot blot for accumulation of vRNA (top panel) and by immunoblot for viral translation/eIF4G cleavage and innate signaling. (**B**) A375 cells were infected with PVSRIPO in the presence of increasing concentrations of the TBK1 inhibitor Bx795, or Bx912 (3 μM), a similarly structured compound with lower TBK1 inhibitory activity than Bx795. Cleavage of eIF4G and viral translation (2BC, 2C) were analyzed by immunoblot. All assays were performed in at least three independent series; representative results are shown.

**
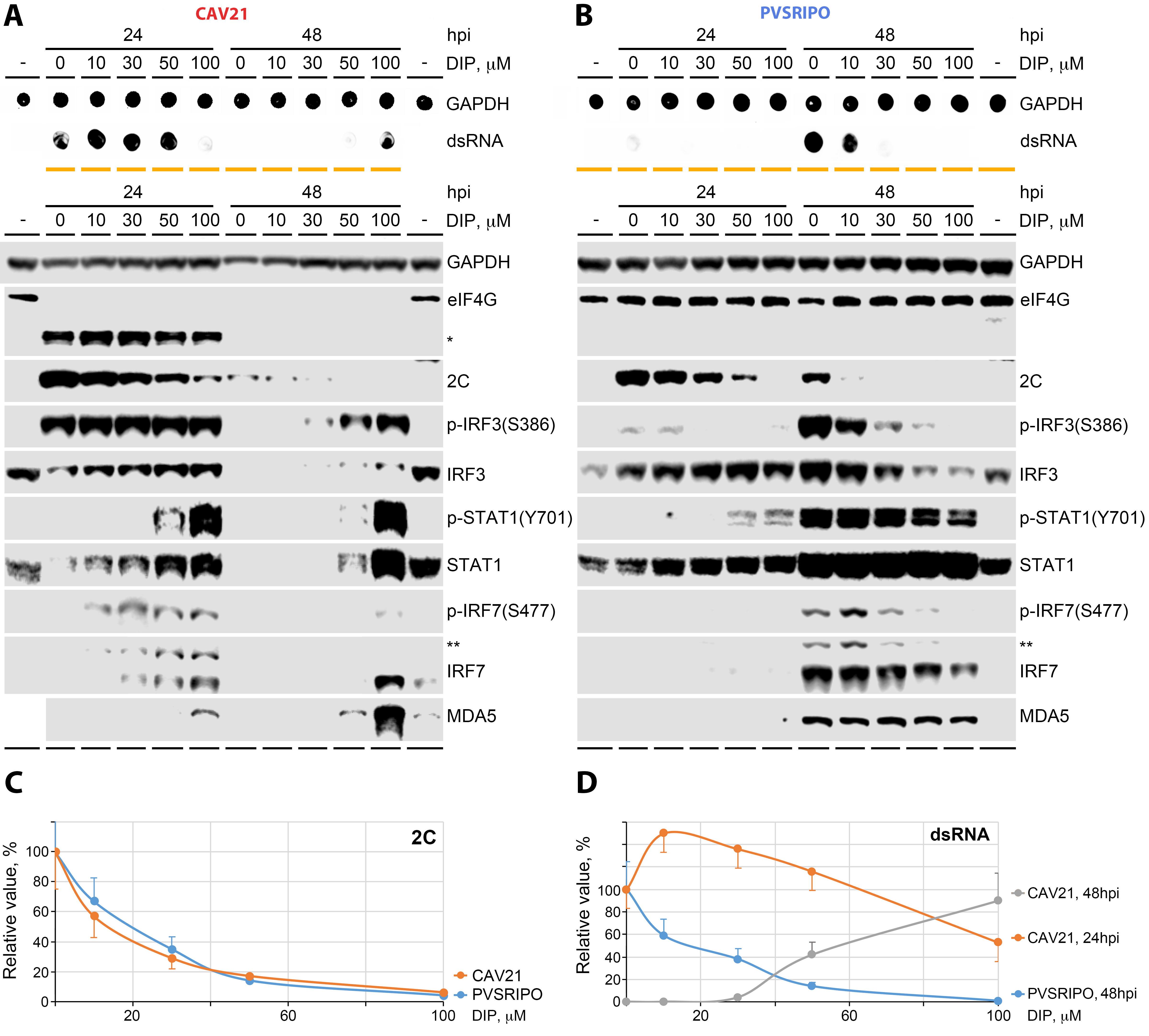
**

**Fig. S5 (related to Figure 6). Dose-titration studies for dipyridamole (DIP).** A375 cells were infected with CAV21 (**A**) or PVSRIPO (**B**), as shown in Figure 2, in the absence (DMSO) or presence of increasing concentrations of DIP. DIP was added at the time of infection (0hpi).

DsRNA accumulation was assessed by dot blot (top panels); viral translation/eIF4G cleavage and type-I IFN responses were tested by immunoblot (bottom panels). All assays were performed in at least 3 independent series. The concentration-dependent effects of DIP on viral translation (2C; **C**) and dsRNA accumulation (**D**) were quantified (%max. normalized to GAPDH; means ^+^/_-_SEM).
